# Development of a novel humanized mouse model for improved evaluation of in vivo anti-cancer effects of anti-PD-1 antibody

**DOI:** 10.1038/s41598-021-00641-8

**Published:** 2021-10-26

**Authors:** Ikumi Katano, Asami Hanazawa, Iyo Otsuka, Takuya Yamaguchi, Misa Mochizuki, Kenji Kawai, Ryoji Ito, Motohito Goto, Takahiro Kagawa, Takeshi Takahashi

**Affiliations:** Laboratory Animal Research Department, Central Institute for Experimental Animals, 3-25-12 Tono-machi, kawasaki-ku, Kawasaki, 210-0821 Japan

**Keywords:** Immunotherapy, Cancer therapy

## Abstract

Immune checkpoint inhibitors (ICIs) have revolutionized the treatment of cancer in the clinic. Further discovery of novel drugs or therapeutic protocols that enhance efficacy requires reliable animal models that recapitulate human immune responses to ICI treatment in vivo. In this study, we utilized an immunodeficient NOG mouse substrain deficient for mouse FcγR genes, NOG-FcγR^−/−^ mice, to evaluate the anti-cancer effects of nivolumab, an anti-programmed cell death-1 (PD-1) antibody. After reconstitution of human immune systems by human hematopoietic stem cell transplantation (huNOG-FcγR^−/−^ mice), four different programmed death-ligand 1 (PD-L1)-positive human cancer cell lines were tested. Among them, the growth of three cell lines was strongly suppressed by nivolumab in huNOG-FcγR^−/−^ mice, but not in conventional huNOG mice. Accordingly, immunohistochemistry demonstrated the enhanced infiltration of human T cells into tumor parenchyma in only nivolumab-treated huNOG-FcγR^−/−^ mice. Consistently, the number of human T cells was increased in the spleen in huNOG-FcγR^−/−^ mice by nivolumab but not in huNOG mice. Furthermore, human PD-L1 expression was strongly induced in the spleen of huNOG-FcγR^−/−^ mice. Collectively, our results suggest that the anti-cancer effects of anti-PD-1 antibodies can be detected more clearly in NOG-FcγR^−/−^ mice than in NOG mice.

## Introduction

The success of immune checkpoint inhibitors (ICIs) such as anti-cytotoxic T-lymphocyte-associated protein-4 (CTLA-4)^1^, anti-programmed cell death-1 (PD-1)^2–4^, and anti-programed death-ligand 1 (PD-L1)^5,6^ antibodies in the clinic have changed the therapeutic strategies for cancer^7–9^. The development of numerous novel compounds or antibodies for combined therapy, which synergistically enhance the anti-cancer effects of anti-CTLA-4 or anti-PD-1 antibodies, is underway^10,11^. Thus, immuno-oncology research has been proceeding toward another medical innovation^12^.

The evaluation of drug efficacy at the preclinical stage requires animal models allowing extrapolation to humans. Humanized mice, which stably and autonomously maintain human tissues, are useful for filling the species gap between humans and animals^13,14^. Immunodeficient mice reconstituted with human hematopoietic and immune systems, in particular, have been extensively used for studying viral infection^15,16^, immune-related diseases such as allergy^17–19^, autoimmune diseases^20,21^, and tumor immunology. Mouse strains highly tolerant to xenogeneic tissues such as NOD/Shi-*scid*-IL2Rγ^*null*^ (NOG)^22^, NOD/LtSz-scidIL-2Rγ^null^ (NSG)^23^, and BALB-RAG2^−/−^ IL-2Rγ^−/−^ double knockout (BRG) mice expressing human signal-regulatory protein α (SIRPA) (BRGS)^24,25^ are frequently used for transplanting human hematopoietic stem cells (huHSCs) or human peripheral blood mononuclear cells (huPBMCs). In these mouse strains, the lack of rearrangement in the B-cell receptor and T-cell receptor genes due to the *scid* mutation or RAG deficiency prevents the development of mature mouse B and T cells. In addition, the targeted disruption of the IL-2Rγ gene results in the absence of NK cells and substantial decreases of lymphoid cells, which require IL-15 and IL-7 signals, respectively. Collectively, these mice show profound immunodeficiency and accept even live human-derived tissues. Moreover, many substrains have also been produced to improve human hematopoiesis and immune functions by introducing various human genes^17,26–30^ or disrupting mouse genes^31^.

For tumor immunology, xenogeneic human cancer cells, either cell line-derived xenograft (CDX) or patient-derived xenograft (PDX), are further transplanted to aforementioned humanized-mice to enable interactions between human immune cells and human tumor cells. Several reports have demonstrated the anti-cancer activities of anti-PD-1, anti-PD-L1, and anti-CTLA-4 antibodies in those co-implantation models^32–37^. Nevertheless, studies on ICIs have often been confounded, since tumor-bearing humanized mice do not show any responses to ICIs in many cases (see “Results” below). Because the response rate to ICI treatment in outpatients in clinics is about 25%^38,39^, resistance to ICIs in humanized mice may reflect the clinical circumstances. Thus, tumor-intrinsic immune suppressive mechanisms in patients may be recapitulated in humanized mice. Alternatively, the absence of expected ICI effects may be attributed to some peculiar features intrinsic to humanized mice: attenuated function of the quasi-acquired immune systems^40^, the development of limited cellular lineages^17^, or interference from mouse innate cells^41^. Therefore, improvement of animal models by identifying key obstacles is critical for the accurate evaluation of ICIs in humanized mice.

In this study, we examined the anti-cancer effects of a therapeutic anti-human PD-1 antibody (nivolumab, OPDIVO; Bristol Myers Squibb™, NY, USA), using a NOG-FcγR^−/−^ mouse, in which FcγR expression is absent due to disruptions of the *Fcer1g and Fcgr2b* genes. As a result, they have minimum capability to induce antibody-dependent cellular cytotoxicity (ADCC) through mouse FcγR^41^. After reconstituting human immune systems by transplanting huHSCs (huNOG-FcγR^−/−^ mice), four different human cancer cells were transplanted. Three of them were effectively rejected by nivolumab treatment in huNOG-FcγR^−/−^ mice, but not in huNOG mice. The rejection was accompanied by the strong infiltration of human T cells into the tumor. These data suggest that NOG-FcγR^−/−^ mice are useful for evaluating the effects of anti-PD-1 antibodies on tumor growth and will help with the development of combination therapies.

## Results

Recently, we established a NOG-FcγR^−/−^ mouse strain, in which the antibody-dependent activation of mouse innate cells is severely suppressed^41^. Many antibody drugs currently under development are based on the human IgG4 isotype because it is considered inert in terms of interactions with human Fcγ receptors. Several reports, however, have shown that interactions between human IgG4 antibodies and mouse Fcγ receptors are possible, and that antibody-dependent biological effects are induced in mouse models^42–44^. Thus, we compared the anti-cancer effects of nivolumab (OPDIVO, a human IgG4) in NOG and NOG-FcγR^−/−^ mice.

### Properties of NOG-FcγR^−/−^ mice in human cell engraftment

**Figure 1:**
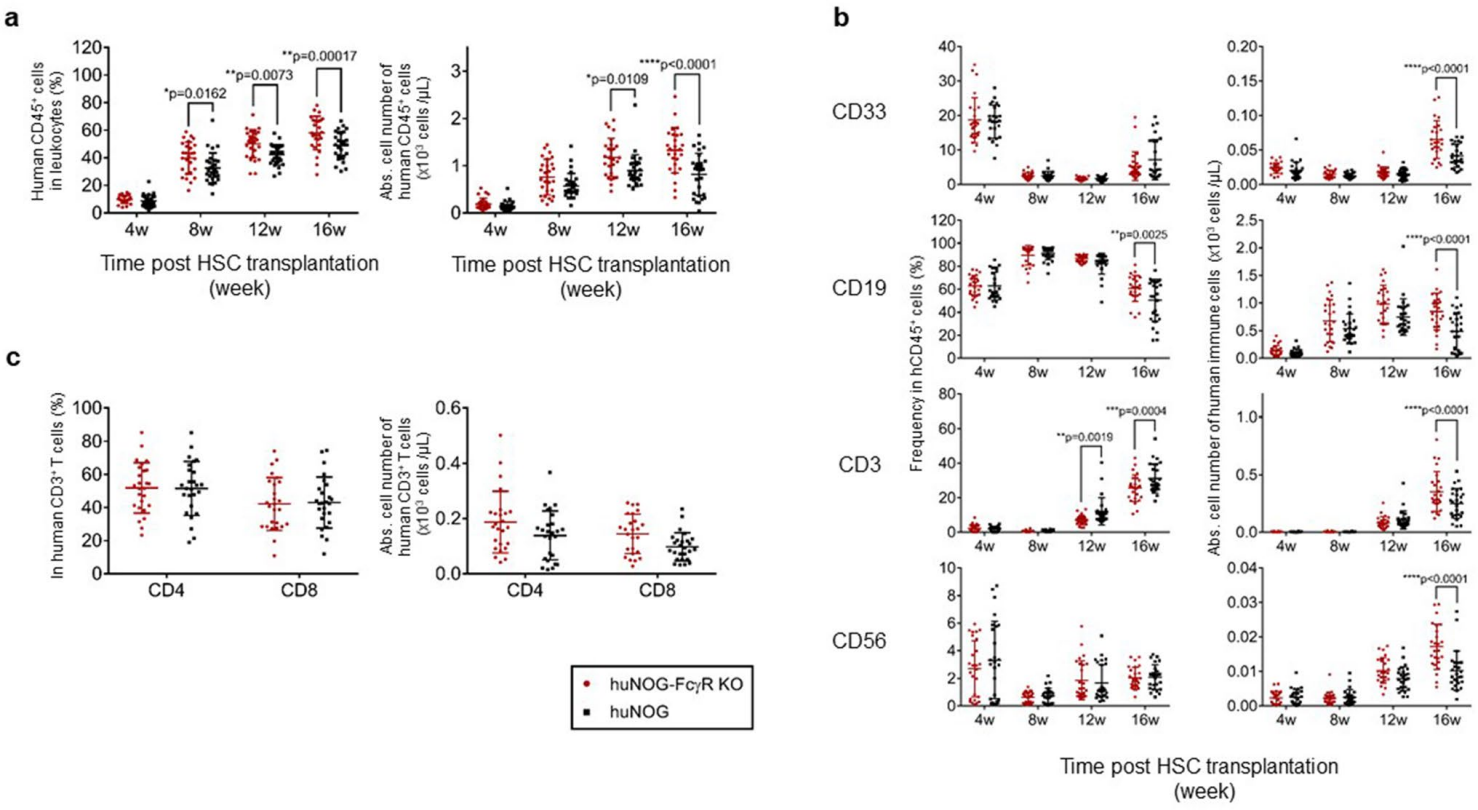
Human hematopoiesis in NOG-FcγR^−/−^ mice. Development of human leukocytes was examined using PB by flow cytometry at the indicated time points. (**a**) Frequency of human CD45^+^ cells in total leukocytes and the absolute cell number. PB was collected from huNOG (black square, n = 28) or huNOG-FcγR^−/−^ mice (red circle, n = 27) at the indicated time points after HSC transplantation and was analyzed by flow cytometry. (**b**) Detection of human CD33^+^ myeloid cells, CD19^+^ B cells, CD3^+^ T cells, and CD56^+^ natural killer (NK) cells. The frequencies in CD45^+^ cells were obtained by flow cytometry analysis and multiplied by the CD45^+^ cell numbers in (**a**) to calculate the cell numbers of each fraction. huNOG (black square, n = 26) or huNOG-FcγR^−/−^ mice (red circle, n = 26). (**c**) Proportion and cell number of CD4^+^ and CD8^+^ T cells. Cumulative data from three independent experiments using HSCs from three different donors are shown with the average and standard deviation (SD) bars. Asterisks indicate statistical significance by a repeated measure ANOVA with Sidak’s multiple comparison test (*p < 0.05, **p < 0.01, ***p < 0.001, ****p < 0.0001).

First, we determined whether huNOG-FcγR^−/−^ mice had any unique immunological features compared with conventional huNOG mice. Analysis of PB from the reconstituted mice demonstrated that the frequency of human CD45^+^ leukocytes in the total leukocytes was higher in huNOG-FcγR^−/−^ mice than in huNOG mice from 8 to 16 weeks post HSC transplantation (wpt) (Fig. 1a). Accordingly, the absolute number of human CD45^+^ cells was higher in huNOG-FcγR^−/−^ mice than in huNOG mice at 12 and 16 wpt (Fig. 1a).

The analysis of human cell subsets demonstrated the significantly higher frequency of CD19^+^ B cells at 16 wpt and lower frequency of CD3^+^ T cells at 12 and 16 wpt in huNOG-FcγR^−/−^ mice than in huNOG mice. There was no difference in CD33^+^ myeloid cells and CD56^+^ natural killer (NK) cells (Fig. 1b). Regarding the absolute cell numbers, there were no significant differences until 12 wpt but all cell fractions became higher in huNOG-FcγR^−/−^ mice than in huNOG mice at 16 wpt (Fig. 1b), reflecting the increase in the total number of human leukocytes. There were no differences in the frequencies and numbers of CD4^+^ and CD8^+^ T cells at 16 wpt (Fig. 1c).

**Figure 2:**
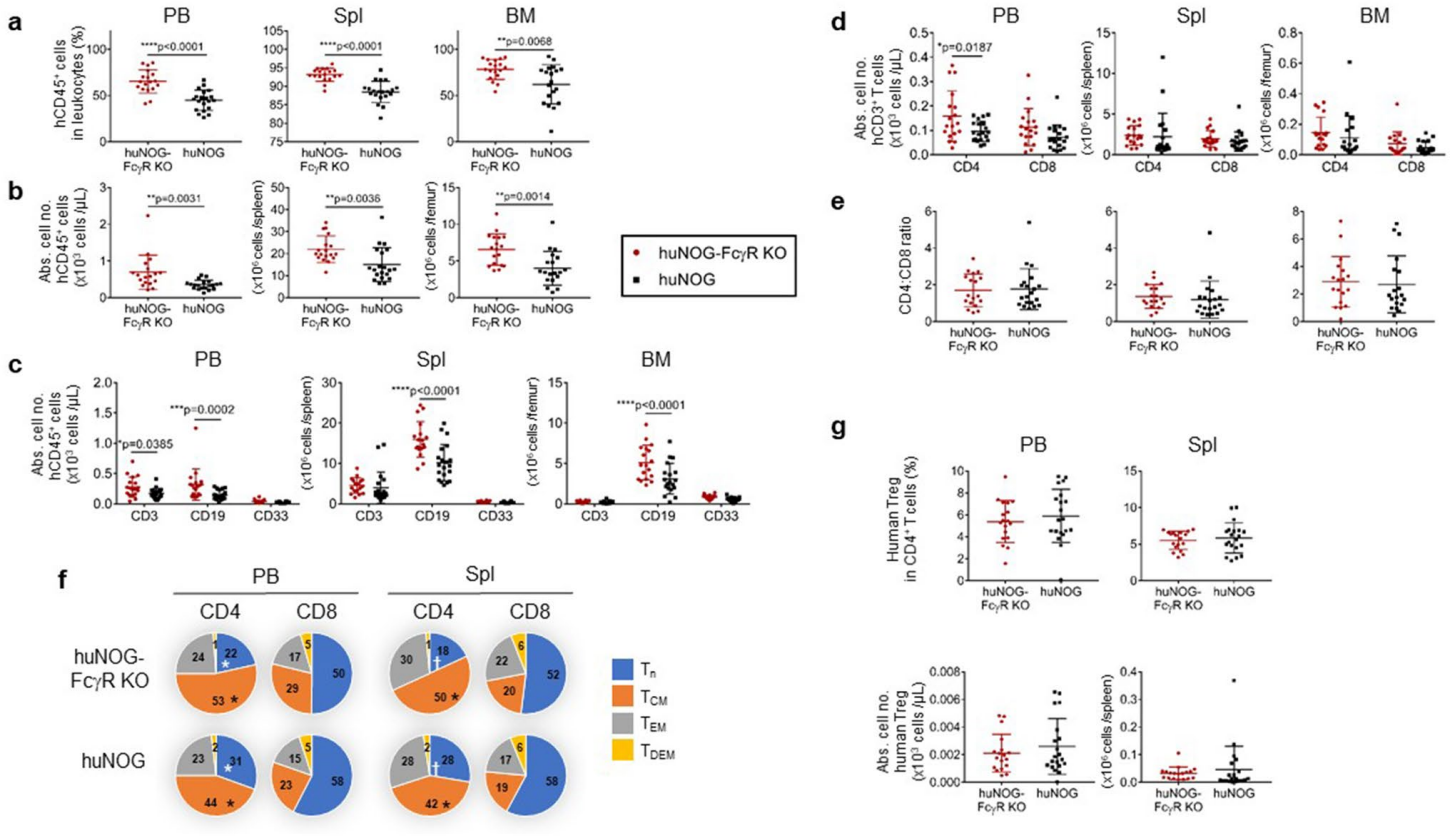
Characteristics of human immune cells in NOG-FcγR^−/−^ mice. Flow cytometry analysis of huNOG and huNOG-FcγR^−/−^ mice at 18 weeks after HSC transplantation. Mononuclear cells from PB, spleen, and BM were examined. (**a**,**b**) The frequencies (**a**) and numbers (**b**) of human CD45^+^ cells in the indicated tissues. Cumulative results from two independent experiments are shown with the average and SD bars. (**c**,**d**) The numbers of human CD3^+^ T cells, CD19^+^ B cells, CD33^+^ myeloid cells (**c**), and T-cell subpopulations (CD4^+^ and CD8^+^) (**d**) are shown. (**e**) Ratios of CD4^+^ to CD8^+^ T cells from (**d**). (**f**) Subpopulations in CD4^+^ and CD8^+^ T cells. T cells were subdivided into T_n_ (CD45RA^+^CD62L^hi^), T_CM_ (CD45RA^-^CD62L^hi^), T_EM_ (CD45RA^-^CD62L^lo^), and T_DEM_ (CD45RA^+^CD62L^lo^) based on the expression of CD45RA and CD62L. The average frequencies of each subpopulation were calculated from flow cytometry data (Supplementary Fig. 2) and depicted in pie charts. (**g**) The frequencies and numbers of human Tregs. Tregs were identified as FOXP3^+^CD25^+^CD45RA^-^CD4^+^ cells detected by intracellular staining^45^ and the data from individual mice are plotted with the average and SD bars. The numbers of huNOG and huNOG- FcγR^−/−^ mice were 20 (19 for BM analysis) and 18, respectively. Asterisks, or a pair of symbols indicate statistical significance in (**a**–**d**), or in (**f**), respectively. The p-value was obtained using two-tailed Student’s *t*-test between huNOG and huNOG-FcγR^−/−^ mice [*p < 0.05, **p < 0.01, ***p < 0.001, ****p < 0.0001 in (**a**–**d**), and *p < 0.05, ^†^p < 0.01 in (**f**)].

Flow cytometry analyses of PB, spleen, and BM was performed at 18 wpt. The frequencies and numbers of human CD45^+^ cells in the total leukocytes were higher in huNOG-FcγR^−/−^ mice than in huNOG mice in all tissues (Fig. 2a,b). Although the frequencies of human CD19^+^ B cells, CD3^+^ T cells, and CD33^+^ myeloid cells were not different between huNOG-FcγR^−/−^ mice and huNOG mice (Supplementary Fig. 1), the absolute number of B cells was significantly higher in huNOG-FcγR^−/−^ mice than in huNOG mice in these tissues (Fig. 2c). By contrast, a significant increase in T-cell number was detected only in PB, which was due to the increase of CD4^+^ T cells (Fig. 2d). The relative ratio of CD4^+^ T cells to CD8^+^ T cells was not different in all tissues between huNOG-FcγR^−/−^ mice and huNOG mice (Fig. 2d, e, Supplementary Fig. 1). There were no significant differences in human myeloid cells (Fig. 2c).

Further fractionation of T cells based on activation status demonstrated that the CD4^+^ naïve T cell (T_n_) population was smaller in huNOG-FcγR^−/−^ mice than in huNOG mice, whereas the proportion of CD4^+^ central-memory T cells (T_CM_) was larger in the former than the latter (Fig. 2f and Supplementary Fig. 2). There were no significant differences in the proportion of CD8^+^ T cell subsets between these two strains (Supplementary Fig. 2). Enumeration of T-cell subpopulations detected a significantly higher number of CD4^+^ T_CM_ cells in PB, and CD8^+^ T_n_ cells in PB in huNOG-FcγR^−/−^ mice (Supplementary Fig. 2). There were no differences in the number of FOXP3^+^CD25^+^CD45RA^-^CD4^+^ regulatory T cells (Tregs)^45^ between huNOG-FcγR^−/−^ mice and huNOG mice (Fig. 2g).

It is common that humanized mouse models with hHSC-reconstitution develop graft vs host disease (GVHD)-like symptoms along with the increase of human T cells. Although the alternation of T- cell subpopulations indicates activation of T cells in huNOG-FcγR^−/−^ mice, the degree of the severity of GVHD in huNOG-FcγR^−/−^ mice was comparable to that in huNOG mice (data not shown).

### Rejection of human tumors by nivolumab in HSC-engrafted NOG-FcγR^−/−^ mice

**Figure 3:**
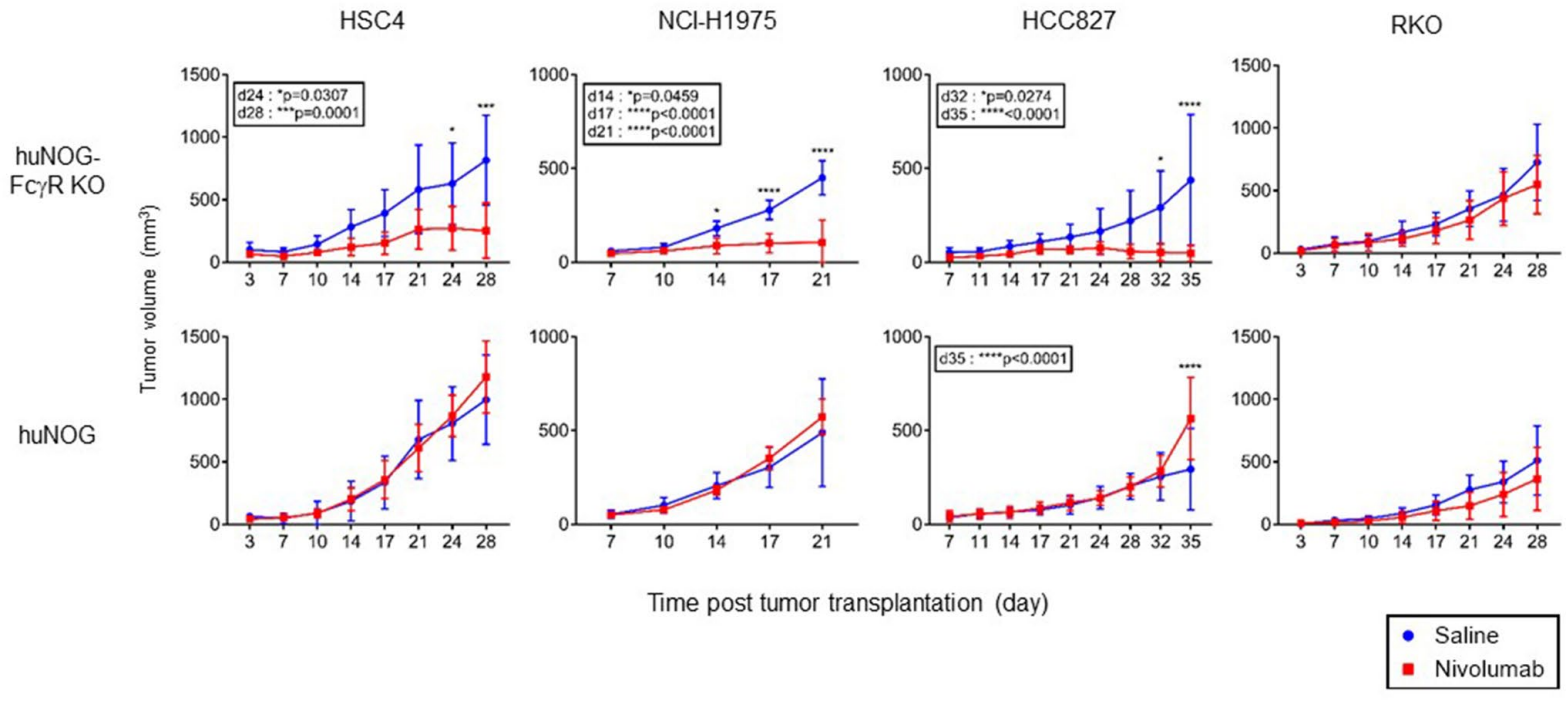
Nivolumab-induced tumor suppression in huNOG-FcγR^−/−^ mice. HSC4, HCC827, NCI-H1975, and RKO cells were subcutaneously inoculated in the flank of huNOG or huNOG-FcγR^−/−^ mice at 12–14 wpt. When the tumor became palpable, 200 μg nivolumab (red line) or saline (blue line) was administered by intraperitoneal (i.p.) injection once a week for 3–4 weeks. The average tumor volume is plotted with SD; the numbers of mice are as follows. For HSC4 and RKO, huNOG mice (n = 5 or 5 for saline- or nivolumab-treated mice, respectively) and huNOG-FcγR^−/−^ mice (n = 4 or 5 for saline- or nivolumab-treated mice, respectively). For NCI-H1975, huNOG mice (n = 5 or 5 for saline- or nivolumab-treated mice, respectively) and huNOG-FcγR^−/−^ mice (n = 5 or 4 for saline- or nivolumab-treated mice, respectively). For HCC827, huNOG mice (n = 5 or 5 for saline- or nivolumab-treated mice, respectively) and huNOG-FcγR^−/−^ mice (n = 4 or 4 for saline- or nivolumab-treated mice, respectively). More than three independent experiments were repeated for each tumor cell line and representative data were shown. Asterisks indicate statistical significance by a repeated measure ANOVA with Sidak’s multiple comparison test. The p-values are indicated in the inset (*p < 0.05, **p < 0.01, ***p < 0.001, ****p < 0.0001).

Next, we compared the anti-tumor effects of nivolumab between huNOG mice and huNOG-FcγR^−/−^ mice. We used a head and neck cancer derived squamous cell carcinoma cell line, HSC4, a colon adenocarcinoma cell line, RKO, and two lung adenocarcinoma cell lines, NCI-H1975 and HCC827. Those tumor cells were subcutaneously transplanted into the flank of huNOG mice and huNOG-FcγR^−/−^ mice at 12–14 wpt, when human T-cell development was confirmed in the PB. Nivolumab was administered once a week for 3–4 weeks after the tumor became palpable. None of the xenogeneic human tumors were suppressed by nivolumab in huNOG mice (Fig. 3), whereas the growth of HSC4, HCC827, and NCI-H1975, but not RKO, was strongly suppressed or even rejected in huNOG-FcγR^−/−^ mice after nivolumab treatment (Fig. 3). In some experiments, tumor growth was enhanced in the nivolumab-treated huNOG mice (HCC827 in Fig. 3). In spite of the strong tumor rejection in nivolumab-treated huNOG-FcγR^−/−^ mice, the formation of tertiary lymphoid structure was not macroscopically detected.

### Infiltration of human T cells in tumors in Nivolumab-treated huNOG-FcγR^−/−^ mice

**Figure 4:**
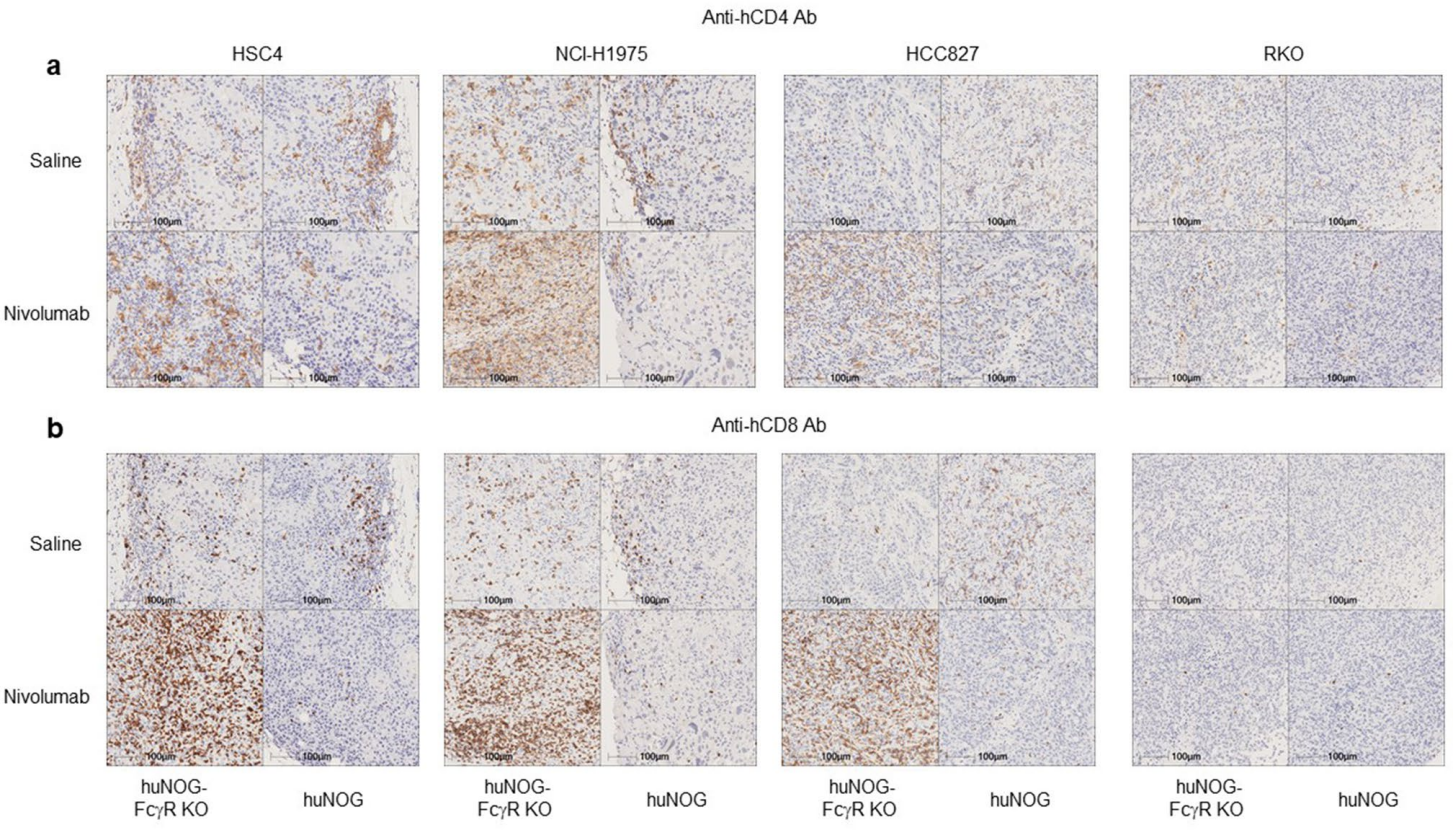
IHC of tumors from huNOG or huNOG-FcγR^−/−^ mice. Tumors were excised from huNOG or huNOG-FcγR^−/−^ mice 1–2 days after the last administration of nivolumab or saline. Half of the tumor was fixed in 10% neutralized formalin and subsequently subjected to IHC analyses after embedding in paraffin. Tumor sections of HSC4, HCC827, NCI-H1975, and RKO cells were stained with anti-human CD4 (**a**) or anti-human CD8 (**b**) antibodies together with hematoxylin for counterstaining. A representative section from a mouse in each group is shown.

**Figure 5:**
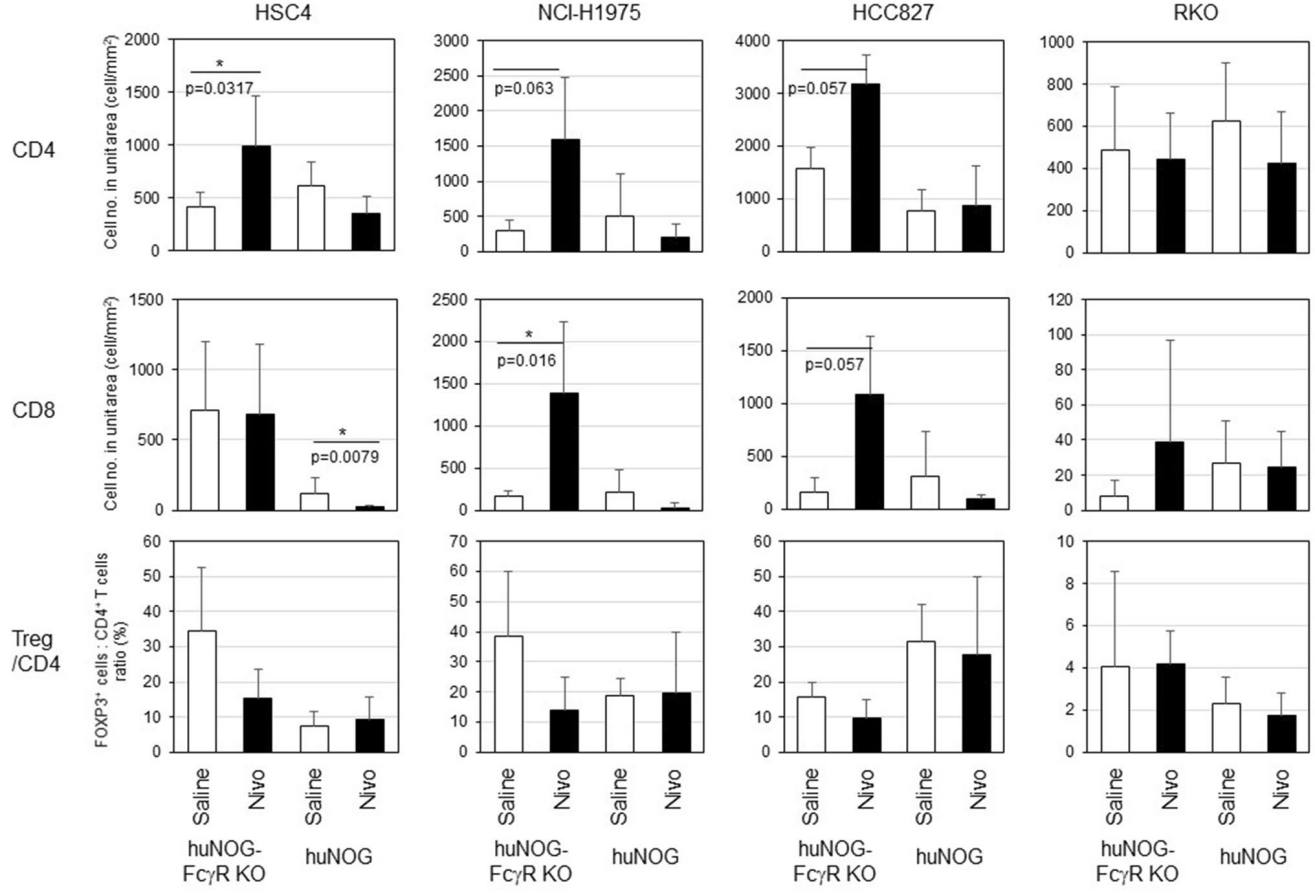
Quantitation of tumor infiltrating human T cells by image analyses. The IHC images in Fig. 4 were used for enumeration of human T cells infiltrating the tumor. The live tumor area was identified by Halo software. T cells in the live tumor area were identified as horseradish peroxidase (brown) and nuclei (blue) double-positive cells. The graphs in the first and second row show the numbers of human CD4^+^ and CD8^+^ T cells per unit live tumor area (T cell number/mm^2^), respectively. The graphs in the bottom row show the ratio of FOXP3^+^ T cells to CD4^+^ T cells in tumors. The means and SD are shown. One representative section was used for the image capture for one mouse and subjected to image analysis. The numbers of mice are as follows. For HSC4, huNOG mice (n = 5 or 5 for saline- or nivolumab-treated mice, respectively) and huNOG-FcγR^−/−^ mice (n = 5 or 5 for saline- or nivolumab-treated mice, respectively). For NCI-H1975, huNOG mice (n = 5 or 5 for saline- or nivolumab-treated mice, respectively) and huNOG-FcγR^−/−^ mice (n = 5 or 4 for saline- or nivolumab-treated mice, respectively). For HCC827, huNOG mice (n = 5 or 5 for saline- or nivolumab-treated mice, respectively) and huNOG-FcγR^−/−^ mice (n = 4 or 3 for saline- or nivolumab-treated mice, respectively). For RKO, huNOG mice (n = 5 or 5 for saline- or nivolumab-treated mice, respectively) and huNOG-FcγR^−/−^ mice (n = 4 or 5 for saline- or nivolumab-treated mice, respectively). Statistical significance was tested by Mann–Whitney *U* test between saline-treated and nivolumab-treated mice in huNOG or huNOG-FcγR^−/−^ mice (*p < 0.05).

The pathological analysis by immunohistochemistry (IHC) demonstrated an enhanced infiltration of human CD4^+^ and CD8^+^ T cells into HSC4, NCI-H1975, and HCC827 tumors, but not in RKO (Fig. 4) in nivolumab-treated huNOG-FcγR^−/−^ mice. By contrast, a few human T cells infiltrated into the tumor in huNOG mice after nivolumab administration (Fig. 4). The quantitation of T cells by image analysis revealed that infiltration of CD4^+^ or CD8^+^ T cells was enhanced by nivolumab treatment in huNOG-FcγR^−/−^ mice, but the degree was largely dependent on the tumor cell lines (Fig. 5). The increase of CD8^+^ T-cell infiltration was statistically significant in NCI-H1975 (Fig. 5). CD4^+^ T cells, but not CD8^+^ T-cell infiltration was enhanced in HSC4 (Fig. 5). In HCC827, there was a substantial increase of both CD4^+^ and CD8^+^ T-cell infiltration, although it did not reach statistical significance. Infiltration of CD8^+^ T cells was remarkably low in RKO, although the number of CD4^+^ T cells was comparable to that in other tumor cell lines. Nivolumab treatment did not enhance infiltration in either huNOG or huNOG-FcγR^−/−^ mice (Fig. 5). Interestingly, there was a tendency that the number of CD8^+^ T cells in the tumor was decreased in nivolumab-treated huNOG mice compared to saline-treated huNOG mice (Fig. 5). Indeed, there was a significant reduction of CD8^+^ T cells in nivolumab-treated HSC4-bearing huNOG mice. FOXP3^+^ Tregs were also detected inside tumor (Supplementary Fig. 3). The ratio of FOXP3^+^ Tregs to CD4^+^ T cells in nivolumab-sensitive cell lines tended to decrease by nivolumab-treatment in huNOG-FcγR^−/−^ mice (Fig. 5). Since the absolute cell number was not decreased (Supplementary Fig. 3), this reduction indicates the infiltration of conventional CD4^+^ T cells into the tumor.

IHC revealed that the localization of human T cells was altered in the spleen of nivolumab-administered huNOG-FcγR^−/−^ mice, but not in huNOG mice (Supplementary Fig. 4). In huNOG mice, there were T-cell clusters in white pulp-like structures irrespective of nivolumab treatment. By contrast, CD8^+^ T cells were diffusively distributed after nivolumab administration in huNOG-FcγR^−/−^ mice (Supplementary Fig. 4), which may indicate the activation of human T cells. In addition, strong PD-L1 expression was induced in the spleen in nivolumab-treated huNOG-FcγR^−/−^ mice, but not in huNOG mice (Supplementary Fig. 5) and some of the PD-L1-positive area corresponded to the CD68-positive area (data not shown). Formation of many strongly PD-L1-positive clusters was evident in the spleen of huNOG-FcγR^−/−^ mice with HSC4 and NCI-H1975 cells. Similar patches were also detected in huNOG-FcγR^−/−^ mice with HCC827 and RKO cells, but the number and size were smaller than in HSC4- or NCI-H1975-bearing mice. Some T cell activation-related cytokines or factors might induce PD-L1 expression in human macrophages. All tumors expressed PD-L1 in control huNOG mice (Supplementary Fig. 6). The expression level was strongly increased in HSC-4- or HCC827-bearing nivolumab-treated huNOG-FcγR^−/−^ mice (Supplementary Fig. 6). The strong expression of PD-L1 in RKO, a resistant cell line, indicates that the rejection of tumors is not always correlated with the expression of PD-L1 in these tumors.

### Influence of nivolumab on human T cells in huNOG and huNOG-FcγR^−/−^ mice

**Figure 6:**
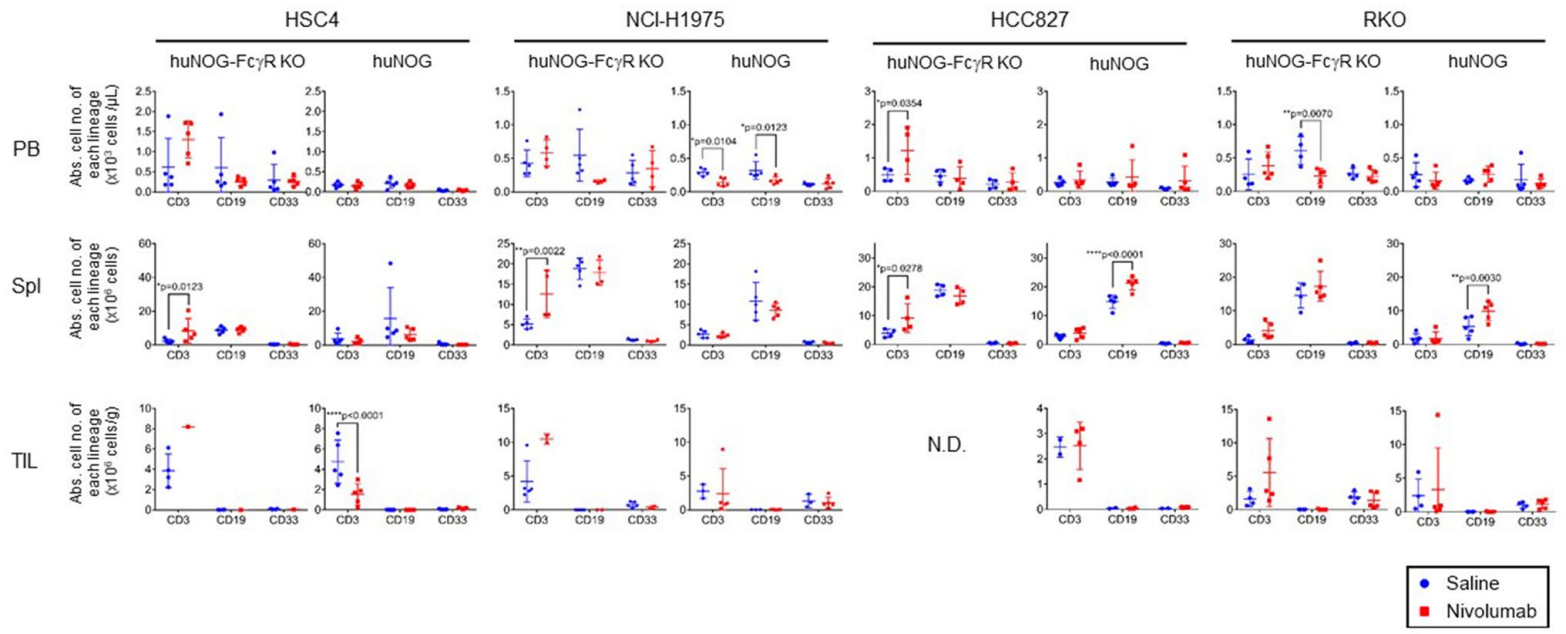
Enumeration of human leukocytes in tumor-bearing humanized mice treated with nivolumab. Mononuclear cells were prepared from the indicated tissues of huNOG or huNOG-FcγR^−/−^ mice. The cells were analyzed by flow cytometry. The absolute cell number of each cell lineage was calculated by multiplying the total cell number with the frequency. Data from a representative experiment were shown for each tumor cell line. The numbers of mice are as follows. For HSC4 and RKO, huNOG mice (n = 5 or 5 for saline- or nivolumab-treated mice, respectively) and huNOG-FcγR^−/−^ mice (n = 4 or 5 for saline- or nivolumab-treated mice, respectively). For NCI-H1975, huNOG mice (n = 5 or 5 for saline- or nivolumab-treated mice, respectively) and huNOG-FcγR^−/−^ mice (n = 5 or 4 for saline- or nivolumab-treated mice, respectively). For HCC827, huNOG mice (n = 5 or 5 for saline- or nivolumab-treated mice, respectively) and huNOG-FcγR^−/−^ mice (n = 4 or 4 for saline- or nivolumab-treated mice, respectively). Note that a fewer number of mice were analyzed for TIL because of the limited tumor size and all tumor tissues were used for IHC analysis (n = 1 or 2 for HSC4- or NCI-H1975-bearing Nivolumab-treated huNOG-FcγR^−/−^ mice, respectively, and n = 3, 2, or 4 for NCI-H1975-, HCC827-, or RKO-bearing saline-treated huNOG mice, respectively). Statistical significance was tested using two-way ANOVA with Sidak’s multiple comparison test (*p < 0.05, **p < 0.01, ***p < 0.001, ****p < 0.0001).

Flow cytometric analyses of human cells in the PB and spleen in tumor-bearing mice demonstrated that the influence of nivolumab treatment was largely dependent on the tumor cell lines and was not the same among mice with different cell lines. However, an increase in the number of human CD3^+^ T cells in the spleen in huNOG-FcγR^−/−^ mice was common in HSC4, NCI-H1975, and HCC827 cells (Fig. 6). This seemed to be mainly due to the increase of CD4^+^ T cells (Supplementary Fig. 8), which was also detected in mice with the nivolumab-resistant RKO.

Analyses of tumor infiltrating lymphocytes (TILs) showed a reduction of human CD3^+^ T cells by nivolumab in HSC4-bearing huNOG mice, which was not evident in other cell lines (Fig. 6). Although TIL analyses by flow cytometry were difficult in nivolumab-treated huNOG-FcγR^−/−^ mice, due to the strong tumor rejection or suppression, some of the nivolumab-treated huNOG-FcγR^−/−^ mice had an elevated number of human CD3^+^ T cells in the tumor compared to control saline-treated huNOG-FcγR^−/−^ mice (Fig. 6). The proportion of CD4^+^ and CD8^+^ T cells was not different between nivolumab-treated mice and control saline-treated mice except for HSC4-bearing huNOG mice, which showed an increase or decrease of CD4^+^ or CD8^+^ T cells, respectively (Supplementary Fig. 7).

Human NK cells were not detected in tumor infiltrating human immune cells by flow cytometry (Supplementary Fig. 9a). The proportion of human monocytes/macrophages was 1 or 5% of human CD8^+^ T cells in nivolumab-treated huNOG-FcγR^−/−^ mice with HSC4 or NCI-H1975, respectively (Supplementary Fig. 9b). These results indicate that T cells are mainly responsible for the tumor rejection.

**Figure 7:**
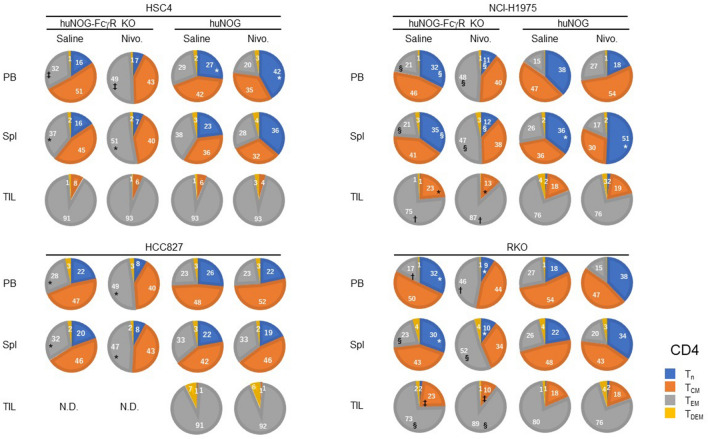
Alternations of CD4^+^ T cell subsets by nivolumab in huNOG-FcγR^−/−^ mice. The proportions of CD4^+^ T cell subsets were obtained from flow cytometry analyses in Fig. 6. The results of the frequencies (Supplementary Fig. 10) and cell numbers (Supplementary Fig. 11) of the T-cell subpopulations are summarized in the pie charts. Data from a representative experiment are shown for each tumor cell line. The value indicates the average. A statistical significance between the corresponding T-cell subsets in saline-treated and nivolumab-treated mice was indicated by a pair of symbols as listed below. Statistical significance was tested using two-way ANOVA with Sidak’s multiple comparison test (*p < 0.05, ^†^p < 0.01, ^‡^p < 0.001, ^§^p < 0.0001).

**Figure 8:**
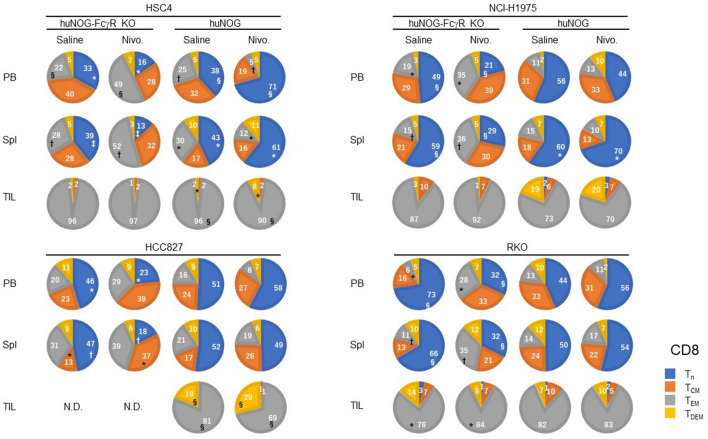
Alternations of CD8^+^ T cell subsets by nivolumab in huNOG-FcγR^−/−^ mice. The proportions of CD8^+^ T cell subsets were obtained from flow cytometry analyses in Fig. 7. The results of the frequencies (Supplementary Fig. 12) and cell numbers (Supplementary Fig. 13) of the T-cell subpopulations are summarized in the pie charts, as in Fig. 7. Statistical significance was tested using two-way ANOVA with Sidak’s multiple comparison test (*p < 0.05, ^†^p < 0.01, ^‡^p < 0.001, ^§^p < 0.0001).

The proportion of T-cell subsets was significantly altered by nivolumab treatment both in huNOG-FcγR^−/−^ and huNOG mice. Although the ways are not the same in different tumor cell lines, an increase of CD4^+^ effector memory T cell (T_EM_) population in the PB and spleen was common in huNOG-FcγR^−/−^ mice (Fig. 7, Supplementary Figs. 10, 11). Accordingly, the size of the CD4^+^ T_n_ population decreased in huNOG-FcγR^−/−^ mice with NCI-H1975 and RKO cells and a similar tendency in mice with HSC4 and HCC827 cells. In huNOG mice, the increase in CD4^+^ T_EM_ was not detected. Rather, an opposite increase of CD4^+^ T_n_ was detected in the PB of HSC4-bearing mice and the spleen of NCI-H1975-bearing mice (Fig. 7). Similar changes were also detected in CD8^+^ T cells. In all of the tumor cell lines, nivolumab induced a decrease of CD8^+^ T_n_ and an increase of activated CD8^+^ T cells in either PB or spleen, or both in huNOG-FcγR^−/−^ mice, T_EM_ for HSC4, NCI-H1975, and RKO or T_CM_ for HCC827 (Fig. 8, Supplementary Figs. 12, 13). On the other hand, nivolumab treatment resulted in an increase of CD8^+^ T_n_ population in the PB and spleen of HSC4-bearing huNOG and in the spleen of NCI-H1975-bearing huNOG mice (Fig. 8). The concomitant decrease of the CD8^+^ T_EM_ population was induced in HSC4-bearing huNOG mice (Fig. 8).

In TILs, most of the CD4^+^ and CD8^+^ T cells had activated phenotypes, and few were naïve T cells in both huNOG-FcγR^−/−^ and huNOG mice prior to nivolumab treatment. The alternations of the composition of CD4^+^ T cell subsets were detected in NCI-H1975- and RKO-bearing huNOG-FcγR^−/−^ mice, namely, a decrease in T_CM_ and increase in T_EM_. No changes in CD4^+^ TIL subsets were detected in huNOG mice after nivolumab treatment (Fig. 7). Regarding CD8^+^ TILs in huNOG-FcγR^−/−^ mice, there were no evident changes except an increase of T_EM_ in RKO-bearing huNOG-FcγR^−/−^ mice (Fig. 8). Interestingly, HSC4- or HCC827-bearing huNOG mice showed a decrease of T_EM_ and increase of terminally differentiated effector-memory T cells (T_DEM_) (Fig. 8).

### Expression of cytokines and cytotoxic molecules in human T cells in huNOG and huNOG-FcγR^−/−^ mice

The analyses of cytokines or cytotoxic molecules in TILs demonstrated that there were considerable differences between tumor cell lines (Supplementary Figs. 14–17). In HSC4, nearly 80% of CD8^+^ T cells in TILs expressed Granzyme B (GZMB) in nivolumab-treated huNOG-FcγR^−/−^ mice, whereas 40% in saline-treated mice (Supplementary Fig. 15). The frequencies of IFN-γ- or TNF-α-positive CD8^+^ T cells were also increased by nivolumab in huNOG-FcγR^−/−^ mice, but not in huNOG mice. Regarding CD4^+^ T cells, the frequencies of GZMB-, IFN-γ-, TNF-α-, or IL-2-expressing TILs were increased by nivolumab in huNOG-FcγR^−/−^ mice (Supplementary Fig. 15). In NCI-H1975, the cytokine expression pattern was different (Supplementary Figs. 14, 16). Although nearly 30–40% of CD8^+^ T cells produced GZMB, IFN-γ, or TNF-α, the frequencies were not changed by nivolumab treatment both in huNOG mice and huNOG-FcγR^−/−^ mice. There were also no clear differences in CD4^+^ TILs (Supplementary Fig. 16). In RKO-engrafted mice, it was difficult to analyze TILs due to the scarcity. In the spleen, the administration of nivolumab substantially decreased the frequencies of GZMB-, TNF-α-, or IL-2-expressing CD4^+^ T cells. But this was detected both in huNOG-FcγR^−/−^ mice and in huNOG mice (Supplementary Fig. 17).

The presence of GZMB-positive T cells in tumor was confirmed by IHC in HSC4-, NCI-H1975-, or HCC827-engrafted huNOG-FcγR^−/−^ mice after nivolumab treatment (Supplementary Fig. 18). Perforin were also detected in TILs, but induction by nivolumab treatment was not as clear as GZMB, especially in HSC4 and HCC827 (Supplementary Fig. 18).

## Discussion

Application of humanized mice to tumor immunology study has been extensively attempted^32–37^, especially for discovering combination drugs synergistically working with ICIs, like an anti-PD-1 antibody. A challenge is the lack of appropriate and reliable animal models that recapitulate human pathology and immune responses. In this study, we demonstrated that the NOG-FcγR^−/−^ mouse strain is more suitable than conventional NOG mice for evaluation of anti-tumor activity of nivolumab.

A unique feature of NOG-FcγR^−/−^ mice is the improved engraftment of human hematopoietic cells. Although the responsible mechanisms are largely unknown and should be clarified, one clue may be that FcR common γ (FcRγ), the product of the fcer1g gene, is a common signal component of multiple receptors. Not only Fc receptors but also immunoglobulin-like transcript (ILT/LIR) receptors (ILT-1, ILT-7, ILT-8, and LIR6a)^46^ or some C type lectin receptors (e.g., Dectin-2, Dectin-3, or Mincle) use FcRγ as an adapter molecule for signaling^47^. Since FcRγ transmits an activating signal through the immunoreceptor tyrosine-based activation motifs (ITAMs), the absence could affect various functions, especially for recognition of foreign substances, in a wide range of mouse innate cells. Indeed, it was reported that neutrophils in FcγR^−/−^ mice mounted a reduced response to integrin signaling^48^, and that mouse platelets require FcγR for the activation by collagen^49^. It is also possible that the absence of FcγRIIb, an inhibitory molecule, alter the functions of mouse innate cells.

Many of the recently developed antibodies including nivolumab have the Fc portion from human IgG4. Evaluation of their functions in conventional mouse models including NOG or NSG mice has been performed based on an assumption that there are no interactions between human IgG4 and mouse FcγRs. Cross-species interactions between human IgG4 and mouse FcγRs, however, can result in biological consequences similar to ADCC in in vivo mouse models^42–44^. Considering the possible interaction of nivolumab and mouse FcγRs, the effective anti-tumor T-cell reactions in huNOG-FcγR^−/−^ mice, but not in huNOG mice, are reminiscent of a previous report by Arlaukas et.al.^50^. The authors showed that tumor-associated macrophage (TAM) deprived tumor-infiltrating CD8^+^ T cells of anti-mouse PD-1 antibody (29F.1A12) on the surface, and that the blocking of mouse FcγRs by specific neutralizing antibodies (2.4G2) induced strong rejection of MC38, a colon adenocarcinoma, in a mouse model^50^. It was suggested that the prolonged presence of anti-PD-1 antibody on the surface of CD8^+^ T cells in the presence of blocking antibody augmented the anti-tumor immune responses. A similar mechanism could function in huNOG-FcγR^−/−^ mice, in which mouse TAMs cannot remove nivolumab on human T cells, resulting in enhanced activation.

Another possibility of evident anti-tumor effects of nivolumab in huNOG-FcγR^−/−^ mice is depletion of human T cells by nivolumab. Our previous report demonstrated that rituximab or trastuzumab significantly suppress the growth of xenogeneic CD20^+^ or HER-2^+^ tumors, respectively, through ADCC in NOG mice, and that growth inhibition is reverted in NOG-FcγR^−/−^ mice^41^. Since human IgG4 can activate mouse macrophages and induce ADCC, it is possible that nivolumab activates mouse macrophages, resulting in the subsequent killing of PD-1^+^ T cells, especially at local tumor sites. Indeed, our HSC4 results demonstrated that nivolumab decreased human T cells in NOG mice, especially at tumor sites, whereas the increase of T cells was oppositely induced in NOG-FcγR^−/−^ mice, which would support this possibility. Finally, the lack of FcγRs might compromise the features of mouse macrophages in NOG-FcγR^−/−^ mice and the immunosuppressive activities in tumor might be attenuated in them, resulting in the activation and expansion of human T cells. Those possible mechanisms are not always mutually exclusive and should be clarified in the future (Supplementary Fig. 19).

Therapeutic anti-PD-1 antibodies block inhibitory signals into T cells, resulting in the augmentation of T-cell activation. This may raise a concern that the reduction of T cells in huNOG mice by nivolumab may simply reflect the downregulation of T-cell receptor (TCR) complexes in activated T cells^51^ or activation induced cell death (AICD) of activated T cells^52^, leading to the failure of detection by flow cytometry. These possibilities, however, seem to be less likely. Since the downregulation of TCR or AICD after antigenic stimulation are T-cell intrinsic features, the same mechanisms would be induced in T cells in huNOG-FcγR^−/−^ mice. Nevertheless, we found an absence of reduction or even increase of human T cells in huNOG-FcγR^−/−^ mice, which suggest that the reduction of T cells in huNOG mice is not caused by TCR downregulation or AICD.

The effective tumor suppression by nivolumab in huNOG-FcγR^−/−^ mice was confirmed in three cell lines. It is interesting, however, that this new mouse model does not assure anti-tumor effects in all cancer cells. Indeed, in addition to RKO, A375 (melanoma, PD-L1 weakly positive), A431 (epidermoid carcinoma, PD-L1 negative), QGP-1 (pancreatic islet cell carcinoma, PD-L1 negative), or A549 (lung adenocarcinoma, PD-L1 weakly positive) were resistant to nivolumab among the cell lines we preliminary tested (data not shown). Human T cells developed in huNOG mice and those human tumor cells are in an allogeneic relationship, since human T cells are positively selected and restricted by mouse MHC in the mouse thymus^40^. Ideally, mouse models like BLT mice, which are engrafted with human fetal thymus and liver tissues together with HSCs from the same donor, may have better allogeneic environment against human cancer xenografts due to the faithful human T-cell selection process provided by the human thymic epithelial cells. Nevertheless, the rejection of HSC4, HCC827, or NCI-H1975 by nivolumab in huNOG-FcγR^−/−^ mice suggests that tumor-derived antigens activate allo-HLA reactive T cells, even if they are restricted by mouse MHC, to invoke anti-tumor immune reactions. Importantly, human T cells seem to be activated by nivolumab in the RKO-bearing mice, since CD4^+^ T cells were increased, and the composition of T cell subpopulations was also changed in the PB and spleen in huNOG-FcγR^−/−^ mice. In addition, PD-L1-strong clusters appeared in the spleen, even though the number was not great compared to HSC4 or NCI-H1975. This raises the question of why those human T cells cannot reject allogenic RKO.

Distribution and cytokine expression in human T cells may give some clues. In HSC4-bearing huNOG-FcγR^−/−^ mice, nivolumab induced both infiltration of T cells into tumor and increases of T-cell subpopulations expressing various cytokines or cytotoxic molecules, which include GZMB, IL-2, or IFN-γ. On the other hand, it was unexpected that nivolumab did not enhance cytokine production in TILs in NCI-H1975-bearing huNOG-FcγR^−/−^ mice, which differed from HSC4-bearing mice. Nevertheless, IHC detected T cells expressing GZMB or Perforin in the tumor in nivolumab-treated huNOG-FcγR^−/−^ mice, which would play a role in rejection of the tumor. Although the results seem to be inconsistent between flow cytometric analysis and IHC, the discrepancy could be attributed to some technical differences in the sample preparation. The low frequencies of T cells with cytokines or cytotoxic molecules were common between RKO-bearing huNOG-FcγR^−/−^ mice and NCI-H1975-bearing mice. However, the exclusion of CD8^+^ T cells from RKO tumor was quite distinct and most likely responsible for the failure of tumor rejection. Although T cells are activated in nivolumab-treated RKO-bearing huNOG-FcγR^−/−^ mice as mentioned above, the functions to infiltrate into the tumor might be compromised as discussed below.

T cells are typically excluded or rendered dysfunctional in developing tumor^53,54^. This has been considered a feature of the tumor microenvironment (TME), and many different types of cellular and molecular mechanisms have been suggested. For example, immune suppressive myeloid cells such as TAMs or myeloid derived suppressor cells (MDSCs) produce immune suppressive molecules such as arginase I, interleukin (IL)-10, or transforming growth factor beta^55,56^. The abnormal vasculature prevents T-cell extravasation and infiltration into tumor^57^. Consumption of nutrients by tumor cells impairs the functions of T cells^58^. Tumor intrinsic mechanisms are also involved. For example, chronic TCR signaling compromises T-cell effector functions^59^. Additionally, tumor cells express several inhibitory molecules such as T-cell immunoglobulin and ITIM (TIGIT), T-cell immunoglobulin and mucin-domain containing-3 (TIM-3), B- and T-lymphocyte attenuator (BTLA), or CD160^60^. Although it should be clarified how the extent of immunosuppressive TME is reconstituted in humanized mice, a study of resistant mechanisms to nivolumab treatment in huNOG-FcγR^−/−^ mice will lead to the discovery of better therapeutic drugs, not only for immunotherapy but also for chemotherapy. Analyses of gene expression in both human and mouse cells constituting the TME and the comparison between susceptible tumors and resistant tumors would provide useful information.

The applications of NOG-FcγR^−/−^ mice will be expanded when they are cross-mated with NOG-human IL-6 (hIL-6) Tg mice^26^ or NOG-hGM-CSF/IL-3 Tg mice^17^. Our group previously reported the enhanced development of human monocytes and TAMs in huNOG-hIL-6 Tg mice, and enhanced myelopoiesis in huNOG-hGM-CSF/IL-3 Tg mice^17^. As mentioned above, TAMs are thought to inhibit T-cell activation in the local tumor environment and be responsible for resistance to various therapies^61^. Thus, NOG-FcγR^−/−^ hIL-6 Tg mice will be useful for studying combination therapies targeting T cells and TAMs. NOG-FcγR^−/−^ hGM-CSF/IL-3 Tg mice will also be useful because of the improved development of human myeloid cells, which may recapitulate human pathological situations in a better way. NOG-class I HLA transgenic mice are also an interesting partner for cross mating with NOG-FcγR^−/−^ mice. Tumor neoantigen-derived peptides have been designed from genome sequence analysis. It is expected that vaccination with those peptides will increase the tumor-specific T cells in an HLA-restricted manner, leading to enhanced recognition and destruction of tumor. NOG-FcγR^−/−^ HLA Tg mice will be useful for the validation.

Taken together, the results of this study demonstrate that NOG-FcγR^−/−^ mice provide a good platform to study anti-tumor T cell responses in the presence of anti-PD-1 antibody. Further sophistication of humanized mouse models through combination with NOG-FcγR^−/−^ mice and other strains will accelerate preclinical experiments and contribute to the discoveries of novel anti-cancer drugs, leading to the improvement of therapeutic strategies and treatment outcomes in the clinic.

## Methods

### Mice

NOG and NOG-FcγR^−/−^ (NOD.Cg-Prkdc^scid^ Il2rg^tm1Sug^ Fcer1g^tm1Rav^ Fcgr2b^tm1Ttk^/Jic) mice^41^ were used in this study. These strains were maintained in the Central Institute for Experimental Animals (CIEA) under specific pathogen-free conditions. All experiments were performed in accordance with institutional guidelines (14038R, 17024, 17025, 20043, and 20044), which were approved by the Animal Experimentation Committee of CIEA. The institutional guidelines are in compliance with the ARRIVE guidelines.

### Transplantation of CD34^+^ human HSCs

For reconstitution of human immune systems, 6- to 8-week old NOG or NOG-FcγR^−/−^ mice were irradiated with 150 cGy X-ray (MBR-1520R-4; Hitachi, Hitachi, Japan) 24 h before transplantation with huHSCs. HuHSCs were purchased from StemExpress (Folsom, CA, USA) and 5 × 10^4^ umbilical cord blood CD34^+^ cells in phosphate-buffered saline (PBS) were given by intravenous injection.

### Cell preparation and flow cytometry

Monoclonal antibodies for staining used in this study are summarized in Supplementary Table 1.

To analyze human cells, multicolor cytometric analyses were performed using a fluorescence-activated cell sorting (FACS) (LSRFortessa X-20™; BD Biosciences, San Jose, CA, USA). To monitor the reconstitution of human cells in NOG or NOG-FcγR^−/−^ mice, PB was collected every 4 weeks to confirm the development of human cells. PB was also assessed using a blood analyzer (LC-662 Microsemi; Horiba, Kyoto, Japan) to enumerate total white blood cells. Red blood cells were lysed using ACK solution (150 mM NH_4_Cl, 10 mM KHCO_3_, 1 mM EDTA-Na_2_) and mononuclear cells (MNCs) were stained with fluorescence-conjugated antibodies. At the time of euthanasia, MNCs were prepared from the PB, spleen, bone marrow (BM), and tumor. MNCs from spleen were prepared by smashing with frosted slide glasses. BM cells were prepared by flushing the femurs with FACS medium [PBS containing 0.5% bovine serum albumin (BSA)] using a 26-gauge needle. Tumor was minced by scissors and subsequently digested in plain RPMI 1640 medium supplemented with type IV collagenase (1 mg/mL; Sigma-Aldrich, St. Louis) and DNase I (100 μg/mL; Roche, Manheim, Germany) for 30 min at 37 °C, and homogenized by a gentleMACS™ homogenizer (Myltenyi Biotec, Bergisch Gladbach, Germany). This step was repeated twice. After digestion, the dissociated cells were filtrated with a cell strainer (70 μm) to remove any clumps. Dead cells were further removed by Debris Removal Solution (Myltenyi Biotec) according to the manufacturer’s instructions and the live cells were suspended with PEB buffer (PBS containing 0.5% BSA and 2 mM EDTA). Those cells were stained with antibody cocktails for 20 min on ice, and subsequently washed with cold FACS medium and subjected to flow cytometry. The data obtained were analyzed by FlowJo software (ver 10.7.1; BD Biosciences).

### Cell culture

The following cell lines were used in this study: HCC827 (lung adenocarcinoma), NCI-H1975 (lung adenocarcinoma) and HSC4 (head and neck squamous cell carcinoma) were from JCRB cell bank (Osaka, Japan). RKO (colorectal carcinoma) was from American Type Culture Collection (Manassas, VA, USA). The cell lines were maintained in complete RPMI 1640 medium supplemented with 10% fetal calf serum with penicillin and streptomycin.

### In vivo tumor transplantation model

Human tumor cells were subcutaneously transplanted into the flank of huNOG or huNOG-FcγR^−/−^ mice at 12–14 weeks post HSC transplantation. The cell number for each cell line per mouse was 1.5 × 10^6^ for HSC4, 2 × 10^6^ for RKO, and 3 × 10^6^ for HCC827 and NCI-H1975. One week after inoculation, when the tumor became visible and palpable, 200 μg anti-PD-1 antibody (nivolumab, OPDIVO) was administered to the mice by intraperitoneal injection once a week for 4 weeks. On the day following the last treatment, the mice were analyzed. Solid tumor size was measured weekly using micrometer calipers and calculated by the following formula: tumor volume [mm^3^] = 1/2 × length (mm) × [width (mm)]^2^.

### Histology

Tumor sections were fixed in 10% neutralized formalin (Mildform; FUJIFILM Wako Pure Chemical, Osaka, Japan). Formalin-fixed tissues were embedded in paraffin and analyzed by IHC. Staining of sections with mouse monoclonal anti-human CD3 (clone: PS1, Nichirei Biosciences Inc., Tokyo, Japan), human CD4 (clone:1F6, Leica Biosystems, Newcastle, UK), human CD8 (clone: 4B11, Leica Biosystems), human FOXP3 (clone: D2W8E, Cell Signaling Technology, MA, USA), or human PD-L1 (clone: 73-10, Abcam, Cambridge, MA, USA) was performed on a fully automated BOND-MAX system (Leica Biosystems, Mount Waverley, VIC, Australia).

The images were captured by a Nanozoomer S60 (Hamamatsu Photonics, Hamamatsu, Japan), and quantitation of positive cells in the images was performed by Halo software (ver. 3.1; Indica labs, Albuquerque, NM, USA) according to the manufacturer’s instructions.

## Supplementary Information

Supplementary Information 1.

Supplementary Information 2.
