# Supplementary material for: Development of a novel humanized mouse model for improved evaluation of in vivo anti-cancer effects of anti-PD-1 antibody
Source: Sci Rep. 2021 Oct 26;11:21087. doi: 10.1038/s41598-021-00641-8 (PMC8548333; doi:10.1038/s41598-021-00641-8)
Supplement: Supplementary file 1 — Supplementary Information 1. [file 41598_2021_641_MOESM1_ESM.docx]

**Title: Development of a novel humanized mouse model for improved evaluation of *in vivo* anti-cancer effects of anti-PD-1 antibody**

Ikumi Katano, Asami Hanazawa, Iyo Otsuka, Takuya Yamaguchi, Misa Mochizuki, Kenji Kawai, Ryoji Ito, Motohito Goto, Takahiro Kagawa, Takeshi Takahashi

**Supplementary Table S1. Antibody list**

| Target | Clone | Fluorescence | Manufacturer |
| --- | --- | --- | --- |
| human CD3 | UCHT1 | Brilliant^TM^ Violet (BV) 421 | BioLegend |
| human CD3 | UCHT1 | Brilliant Ultraviolet (BUV)737 | BD Biosciences |
| human CD4 | RPA-T4 | FITC | BioLegend |
| human CD4 | RPA-T4 | Phycoerythrin/Cyanine (PE/Cy) 7 | BioLegend |
| human CD4 | RPA-T4 | Allophycocyanin/Cyanine (APC/Cy) 7 | BioLegend |
| human CD4 | OKT4 | BV421 | BioLegend |
| human CD8a | RPA-T8 | APC | BioLegend |
| human CD8a | RPA-T8 | BUV395 | BD Biosciences |
| human CD14 | M5E2 | BUV737 | BD Biosciences |
| human CD16 | 3G8 | BV 605 | BioLegend |
| human CD19 | HIB19 | PE/Cy7 | BioLegend |
| human CD25 | 2A3 | Brilliant Blue 515 | BD Biosciences |
| human CD33 | WM-53 | BUV395 | BD Biosciences |
| mouse CD45 | 30-F11 | APC/Cy7 | BioLegend |
| human CD45 | HI30 | BV 510 | BioLegend |
| human CD45RA | HI100 | BUV737 | BD Biosciences |
| human CD56 | NCAM16.2 | APC-R700 | BD Biosciences |
| human CD62L | DREG-56 | PE/Cy7 | BioLegend |
| human Foxp3 | PCH101 | PE | eBioscience |
| human Granzyme B | QA16A02 | FITC | BioLegend |
| human IFN-γ | 4S.B3 | PE | BioLegend |
| human IL-2 | MQ1-17H12 | PE/Cy7 | BioLegend |
| human TNF-α | MAb11 | PerCP/Cy5.5 | BioLegend |
| Mouse IgG1, κ Isotype Ctrl. | MOPC-21 | FITC | BioLegend |
| Mouse IgG1, κ Isotype Ctrl. | MOPC-21 | PE | BioLegend |
| Mouse IgG1, κ Isotype Ctrl. | MOPC-21 | PerCP/Cy5.5 | BioLegend |
| Rat IgG2a, κ Isotype Ctrl. | RTK2758 | PE | BioLegend |
| Rat IgG2a, κ Isotype Ctrl. | RTK2758 | PE/Cy7 | BioLegend |
| Ultra-LEAF^TM^ Purified anti-mouse CD16/32 | 93 | - | BioLegend |
| Human TruStain FcX^TM^ | - | - | BioLegend |
|  |  |  |  |
|  |  |  |  |
|  |  |  | BD Biosciences, San Jose, CA |
|  |  |  | Biolegend, San Diego, CA |
|  |  |  | eBioscience, San Diego, CA |
|  |  |  | Santa Cruz Biotechnology, Dallas, TX |

**Supplementary Figure Legends**

**Figure S1. Frequency of human leukocyte subsets**

Flow cytometry analyses of PB, spleen, and BM from huNOG or huNOG-FcγR^-/-^ mice at 18 wpt. The frequencies of human B cells, T cells, and myeloid cells in total human CD45^+^ leukocytes are shown (a). The frequencies of CD4^+^ and CD8^+^ T cells in total human CD3^+^ T cells are shown (b).

**Figure S2. Frequency and number of human T-cell subpopulations**

The frequencies and the absolute cell numbers are shown for T_n,_ T_CM_, T_EM_, and T_DEM_ cells in CD4^+^ and CD8^+^ T cells in PB and spleen of huNOG or huNOG-FcγR^-/-^ mice at 18 wpt. Statistical significance was tested using two-way ANOVA with Sidak’s multiple comparison test (* p < 0.05, ** p < 0.01, and *** p < 0.001, ns: no significance).

**Figure S3. IHC analyses of FOXP3^+^ Treg cells in tumor**

Tumor sections in Figure 5 were stained with anti-human FOXP3 antibody. Representative images are shown. Bar graphs show the cell number of FOXP3^+^ cells per unit live tumor area (cell number/mm^2^). The average with SD bars are shown. Statistical significance was tested by Mann–Whitney U test between saline-treated and nivolumab-treated mice in huNOG or huNOG-FcγR^-/-^ mice (* p < 0.05).

**Figure S4. IHC analyses of CD8^+^ T cells in spleen**

Spleen was collected from huNOG or huNOG-FcγR^-/-^ mice. Half of the tissue was fixed in 10% neutralized formalin and subsequently subjected to the immunohistochemical analysis as in Figure 4. Representative images of IHC with an anti-CD8 antibody are shown. A representative section was shown for each mouse group.

**Figure S5. IHC analysis of human PD-L1 in the spleen of huNOG or huNOG-FcγR^-/-^ mice.**

Spleen from huNOG or huNOG-FcγR^-/-^ mice were stained with an anti-human PD-L1 antibody with hematoxylin. A representative image of sections is shown. Bar graphs show the average percentages of hPD-L1-positive area to total tissue area, which was determined by image analysis. A representative section was used for the image capture for each mouse. Statistical significance was tested by Mann–Whitney U test between saline-treated and nivolumab-treated mice in huNOG or huNOG-FcγR^-/-^ mice (* p < 0.05, ** p < 0.01).

**Figure S6. IHC analyses of PD-L1 expression in tumor**

Tumor sections prepared from huNOG or huNOG-FcγR^-/-^ mice were subjected to IHC analysis by anti-human PD-L1 antibody. A representative image is shown for each mouse group.

**Figure S7. Frequencies of CD4^+^ and CD8^+^ T cells in tumor-engrafted huNOG and huNOG-FcγR^-/-^ mice**

Results from flow cytometry analyses of mononuclear cells from the mice in Figure 6. The frequencies of human CD4^+^ and CD8^+^ T cells in human CD3^+^ T cells are shown. Statistical significance was tested using two-way ANOVA with Sidak’s multiple comparison test (* p < 0.05, ns: no significance).

**Figure 8. Cell numbers of CD4^+^ and CD8^+^ T cells in tumor-engrafted huNOG and huNOG-FcγR^-/-^ mice**

The numbers of human CD4^+^ and CD8^+^ T cells in PB (per unit volume, /μL), spleen and TILs (per unit weight, /gram) are shown. Statistical significance was tested using a two-way ANOVA with Sidak’s multiple comparison test (* p < 0.05, ** p < 0.01, ns: no significance).

**Figure S9. Frequencies of human NK cells and monocytes/macrophages in tumor-infiltrating human immune cells in huNOG-FcγR^-/-^ mice**

Results from flow cytometry analyses of tumor-infiltrating human immune cells from the mice in Figure 6. (a) The frequencies of human CD56^+^ NK cells in human CD45^+^ leukocytes are shown. (b) Ratios of human CD14^+^ monocytes and macrophages to human CD8^+^ T cells are shown. Statistical significance was determined with Mann-Whitney U test. (* p < 0.05 and ** p < 0.01).

**Figure S10.** **Frequencies of T-cell subpopulations in CD4^+^ T cells in tumor-engrafted huNOG and huNOG-FcγR^-/-^ mice**

Results from flow cytometry analyses of mononuclear cells from the mice in Figure 7. The frequencies of human CD4^+^ T_n_, T_CM_, T_EM_, and T_DEM_ are shown. Statistical significance was tested using two-way ANOVA with Sidak’s multiple comparison test (* p < 0.05, ** p < 0.01, *** p < 0.001, **** p < 0.0001).

**Figure S11.** **Cell numbers of T-cell subpopulations in CD4^+^ T cells in tumor-engrafted huNOG and huNOG-FcγR^-/-^ mice**

The absolute cell numbers of human CD4^+^ T_n_, T_CM_, T_EM_, and T_DEM_ are shown. Statistical significance was tested using two-way ANOVA with Sidak’s multiple comparison test (* p < 0.05, ** p < 0.01, *** p < 0.005, **** p < 0.0001).

**Figure S12.** **Frequencies of T-cell subpopulations in CD8^+^ T cells in tumor-engrafted huNOG and huNOG-FcγR^-/-^ mice**

Results from flow cytometry analyses of mononuclear cells from the mice in Figure 8. The frequencies of human CD8^+^ T_n_, T_CM_, T_EM_, and T_DEM_ are shown. Statistical significance was tested using two-way ANOVA with Sidak’s multiple comparison test (* p < 0.05, ** p < 0.01, *** p < 0.001, **** p < 0.0001).

**Figure S13.** **Cell numbers of T-cell subpopulations in CD8^+^ T cells in tumor-engrafted huNOG and huNOG-FcγR^-/-^ mice**

The absolute cell numbers of human CD8^+^ T_n_, T_CM_, T_EM_, and T_DEM_ are shown. Statistical significance was tested using two-way ANOVA with Sidak’s multiple comparison test (* p < 0.05, ** p < 0.01, *** p < 0.005, **** p < 0.0001).

**Figure S14. Intracellular staining of cytokines in TILs in tumor-bearing huNOG-FcγR^-/-^ mice**

Cells were restimulated with 50 ng/mL PMA (Sigma-Aldrich) and 1 μg/mL ionomycin (Sigma-Aldrich) in the presence of 3 μg/mL Brefeldin A (Biolegend, San Diego, CA) for 4 h at 37^o^C, except for Granzyme B (GZMB), for which cells were restimulated for 20h. The cells were recovered and stained with Live Dead solution (Invitrogen, Waltham, MA) for 30 min on ice. After washing, the Fc receptors were blocked by anti-mouse CD16/32 antibodies and Human FcX true stain. The surface markers were stained with anti-hCD4-Brilliant Violet (BV)-421, anti-hCD45-BV-510, anti-CD8a-Brilliant Ultraviolet (BUV)-395, and anti-CD3-BUV737 antibodies. The cells were washed with FACS buffer and then fixed with the Fixation Buffer for 20 min at room temperature (RT) in the dark (eBioscience). The cells were permeabilized with Intracellular Staining Permeabilization Wash Buffer (Biolegend) and incubated with 5% normal mouse serum (Invitrogen) for 20 min at RT for intracellular blocking. Intracellular molecules were stained for 30 min with the antibody cocktails as shown below. Those cells were analyzed by flow cytometer after the final wash with permeabilization buffer.

|  | Fluorescence | Cocktail-1 | Cocktail-2 | Cocktail-3 |
| --- | --- | --- | --- | --- |
| Abs | FITC |  | hGranzyme B | Isotype Ab |
|  | PE | hIFNγ |  | Isotype Ab |
|  | PerCP/Cy5.5 | hTNF-α |  | Isotype Ab |
|  | PE/Cy7 | hIL-2 |  | Isotype Ab |

**Figure S15. Cytokine production in T cells from HSC4-engrafted humanized mice**

Production of cytokines or cytotoxic molecules in TILs was analyzed as described in Figure S14. (n = 3 or 3 for HSC4-bearing saline- or Nivolumab-treated huNOG-FcγR^-/-^ mice, respectively, and n = 2 or 2 for HSC4-bearing saline- or Nivolumab-treated huNOG, respectively). A representative result from two independent experiments is shown.

**Figure S16. Cytokine production in T cells from NCI-H1975-engrafted humanized mice**

Production of cytokines or cytotoxic molecules in TILs was analyzed as described in Figure S14. (n = 3 or 2 for NCI-H1975-bearing saline- or Nivolumab-treated huNOG-FcγR^-/-^ mice, respectively, and n = 3 or 3 for NCI-H1975-bearing saline- or Nivolumab-treated huNOG, respectively).

**Figure S17.** **Cytokine production in T cells from RKO-engrafted humanized mice**

Production of cytokines or cytotoxic molecules in splenic T cells (n = 2 or 2 for RKO-bearing saline- or Nivolumab-treated huNOG-FcγR^-/-^ mice, respectively, and n = 3 or 3 for RKO-bearing saline- or Nivolumab-treated huNOG, respectively). TIL analysis was impossible for this cell line due to the scarce number of infiltrating human T cells.

**Figure S18. IHC of cytotoxic molecules in TILs**

Tumor sections were stained with anti-human Granzyme B (clone: 11F1, Leica Biosystems) or anti-human Perforin (clone: 5B10, Invitrogen) antibodies together with hematoxylin. A representative section from a mouse in each group is shown.

**Figure S19. Schematic mechanisms for T-cell activation by nivolumab in huNOG-FcγR^-/-^ mice.**

Possible mechanisms for the absence of anti-cancer effects by nivolumab in huNOG mice (left). Genetic depletion of mouse FcγR restores nivolumab effects, resulting in the T-cell activation in huNOG-FcγR^-/-^ mice (right).
