# Supplementary material for: Development of a novel humanized mouse model for improved evaluation of in vivo anti-cancer effects of anti-PD-1 antibody
Source: Sci Rep. 2021 Oct 26;11:21087. doi: 10.1038/s41598-021-00641-8 (PMC8548333; doi:10.1038/s41598-021-00641-8)
Supplement: Supplementary file 2 — Supplementary Information 2. [file 41598_2021_641_MOESM2_ESM.pdf]

Title: Development of a novel humanized mouse model for improved evaluation of in vivo anti-cancer effects of anti-PD-1 antibody

Ikumi Katano, Asami Hanazawa, Iyo Otsuka, Takuya Yamaguchi, Misa Mochizuki, Kenji Kawai, Ryoji Ito, Motohito Goto, Takahiro Kagawa, Takeshi Takahashi

Supplementary Figure 1

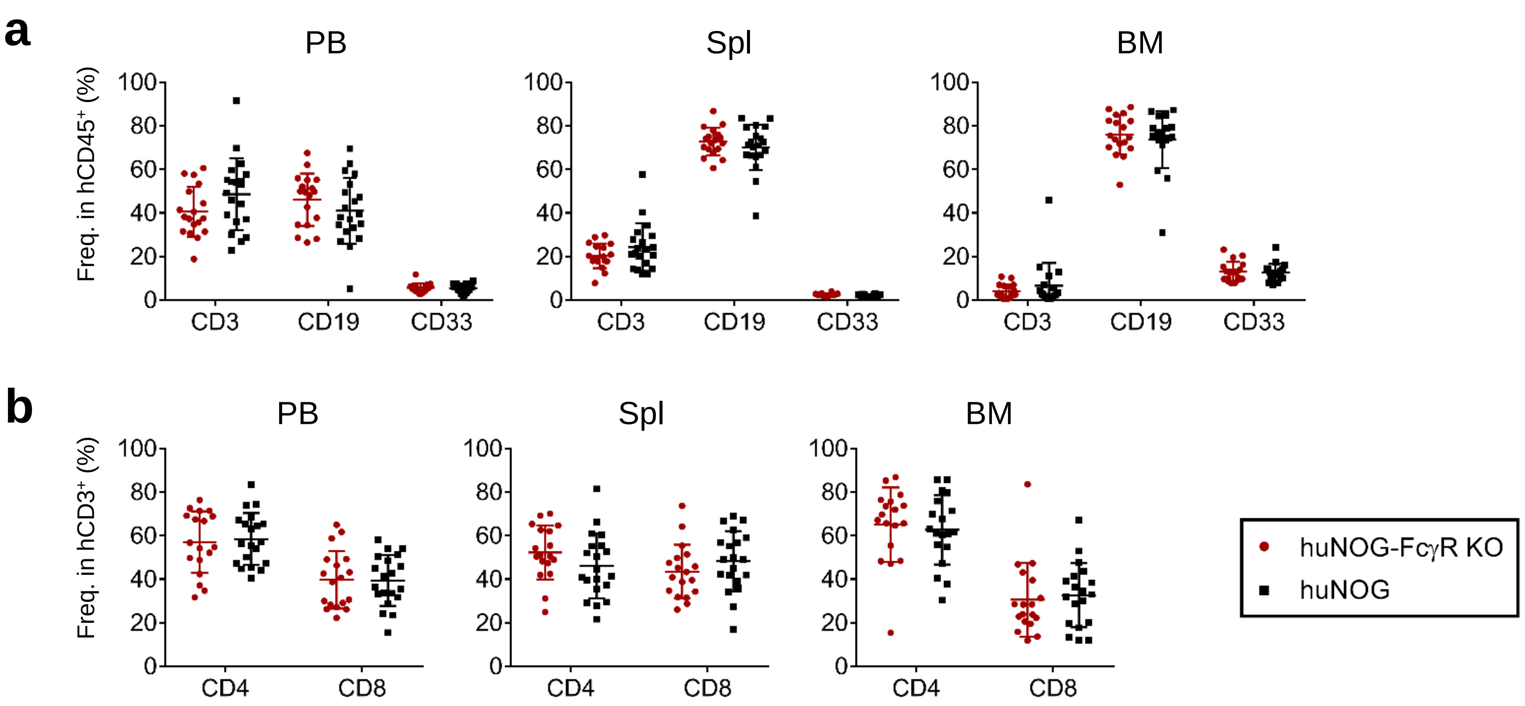

Supplementary Figure 2

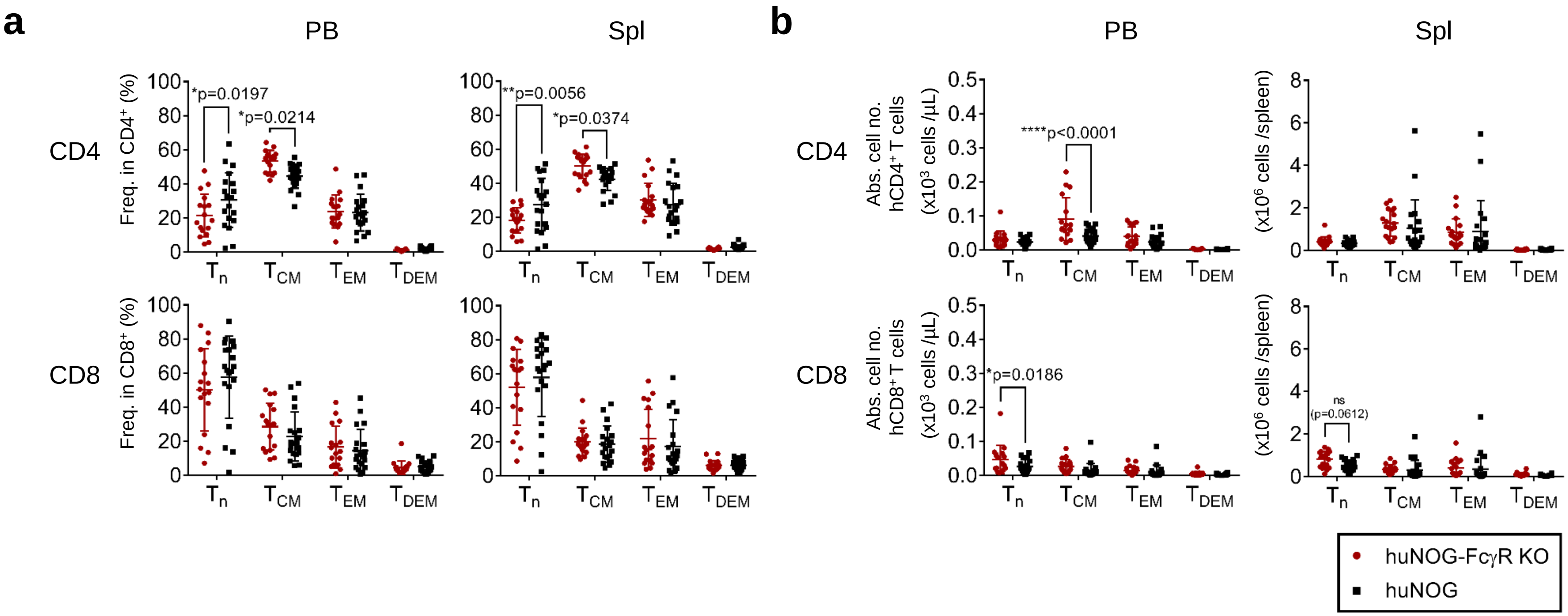

Supplementary Figure 3

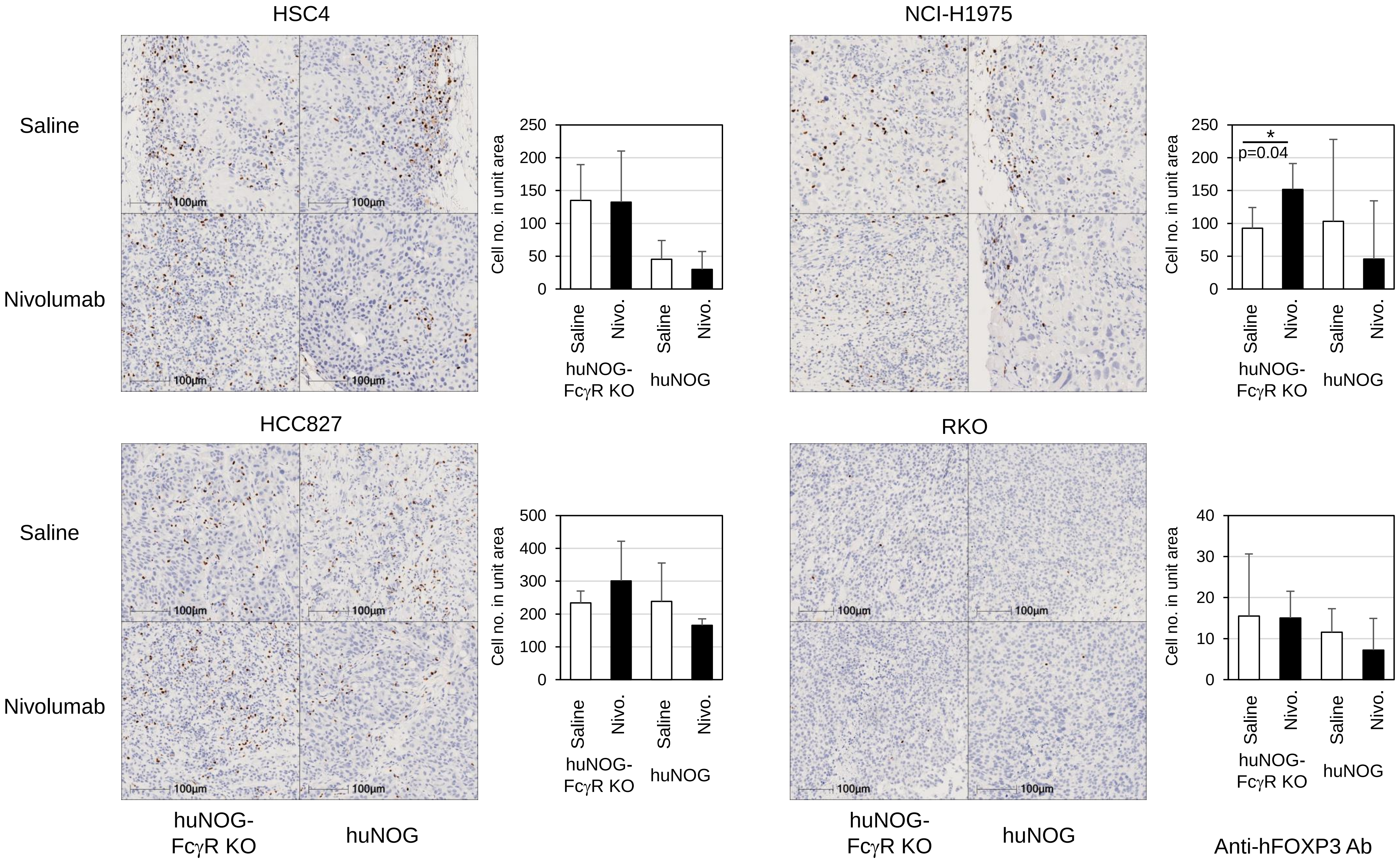

Supplementary Figure 4

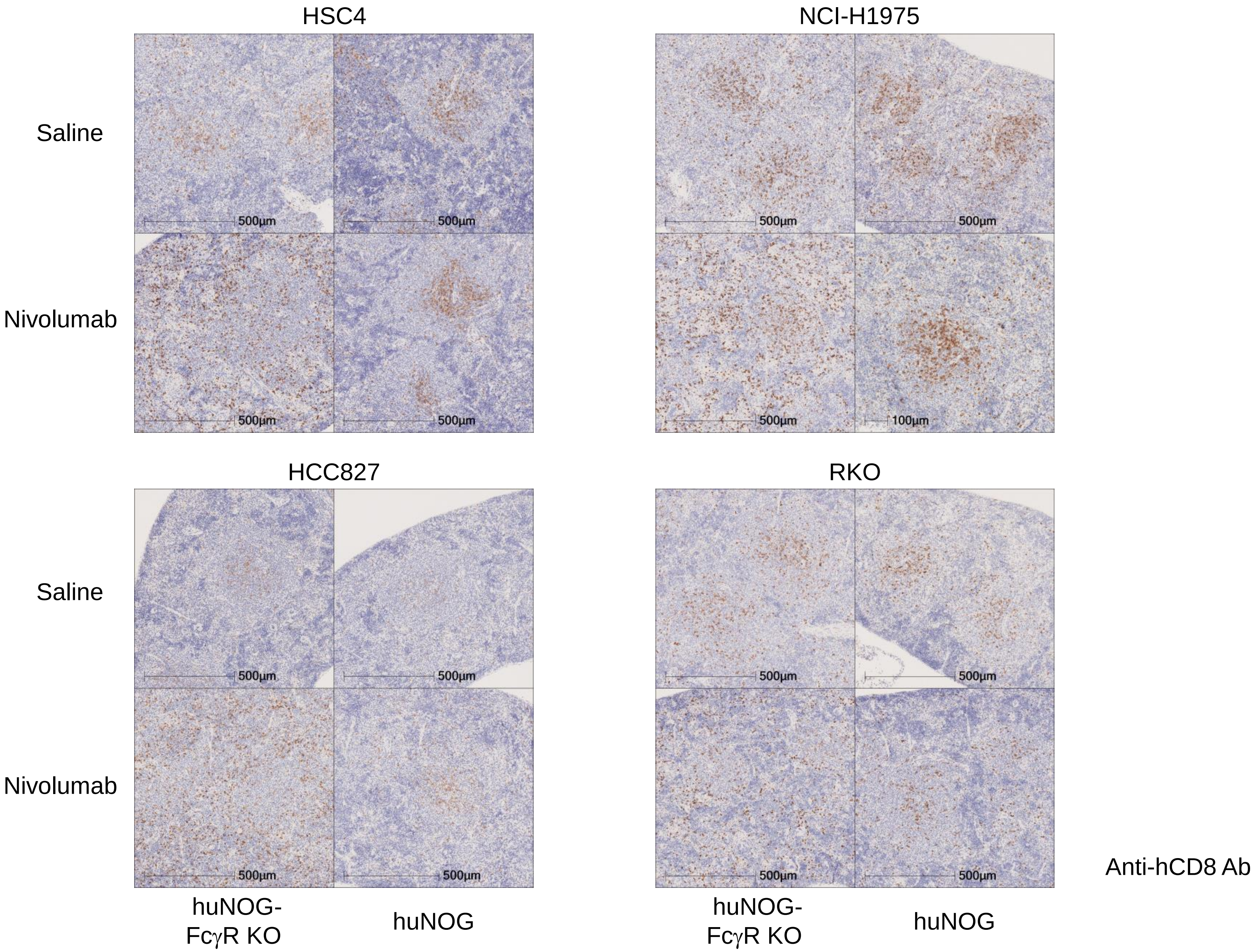

Supplementary Figure 5

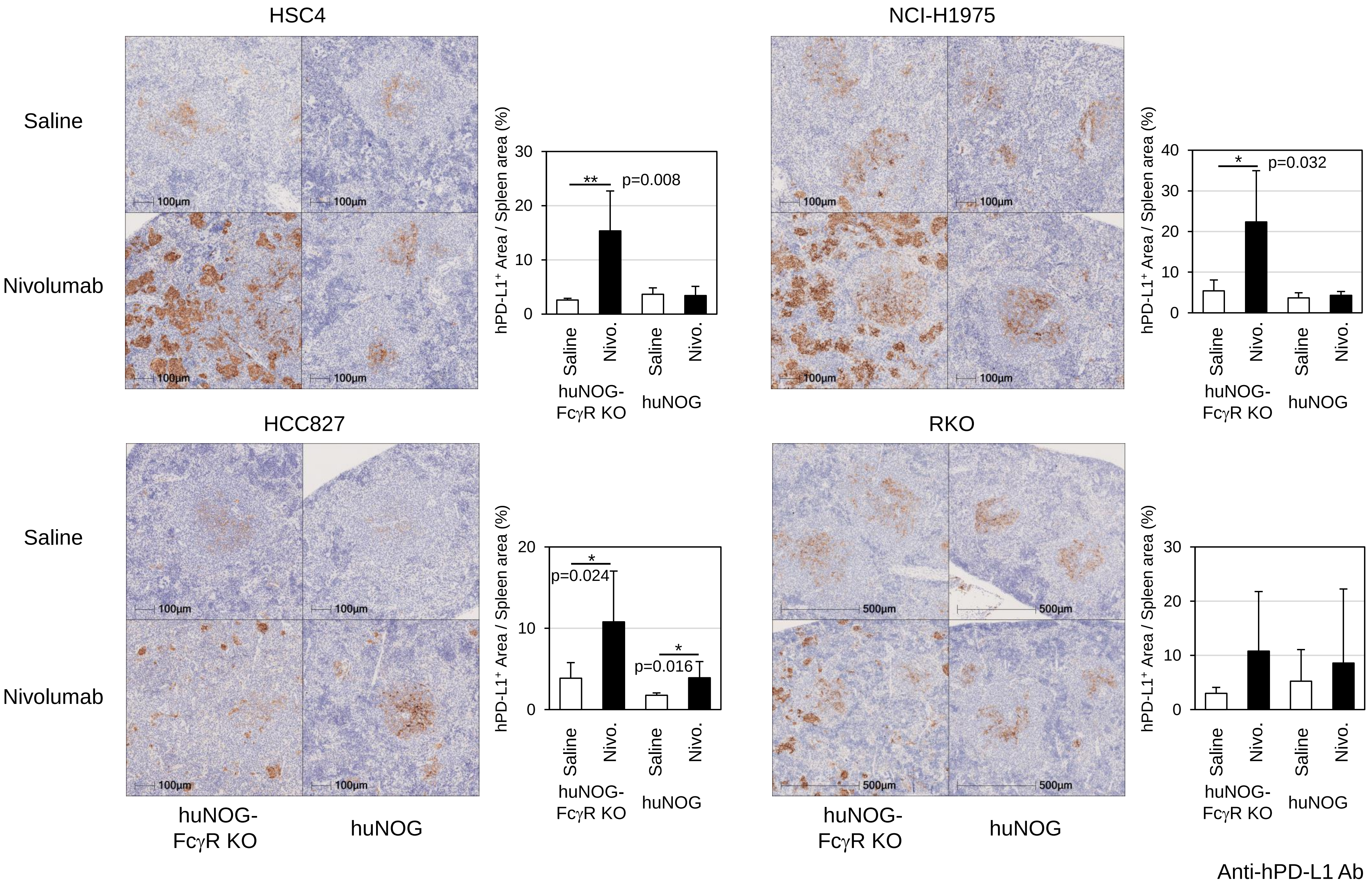

Supplementary Figure 6

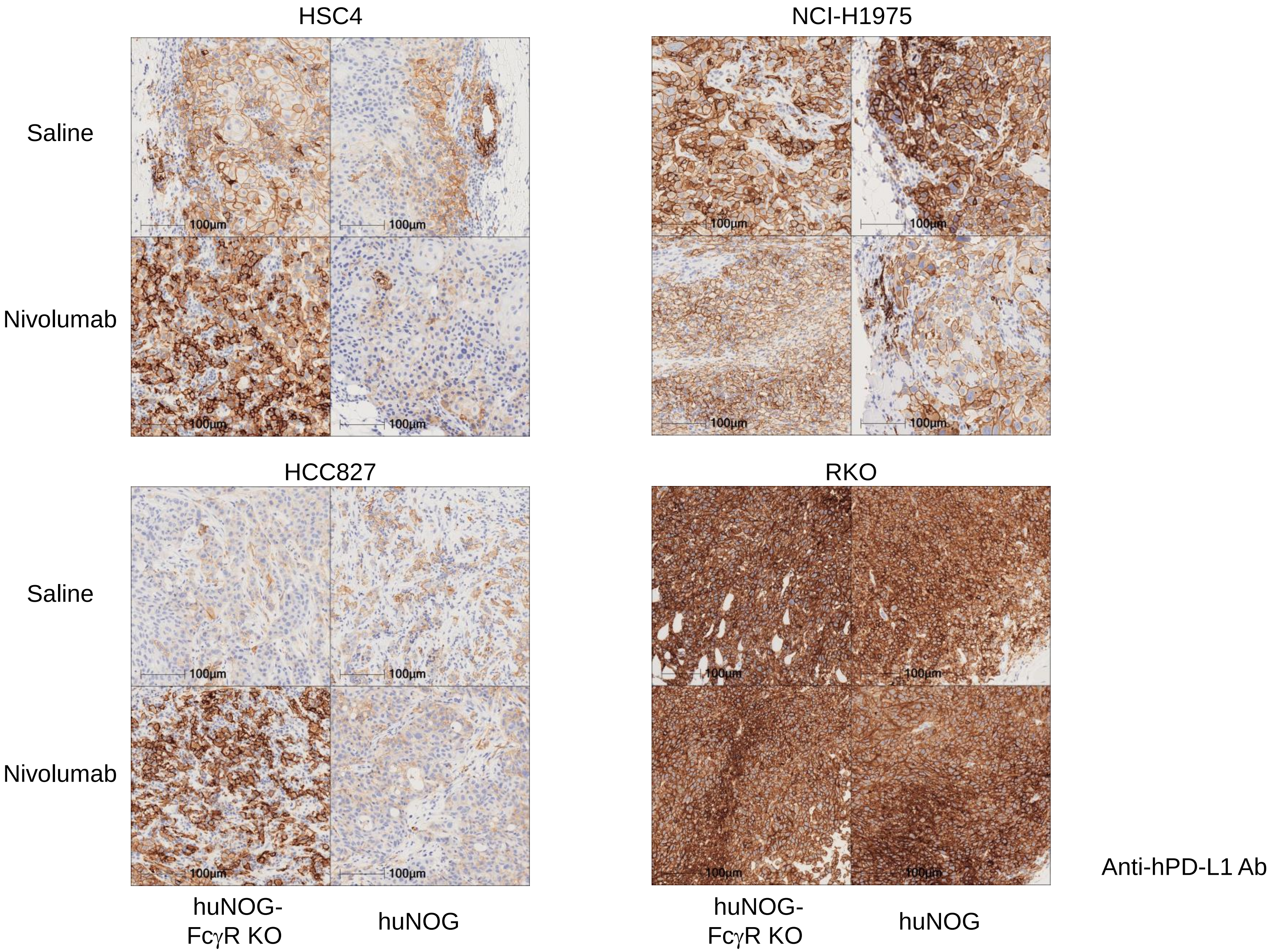

Supplementary Figure 7

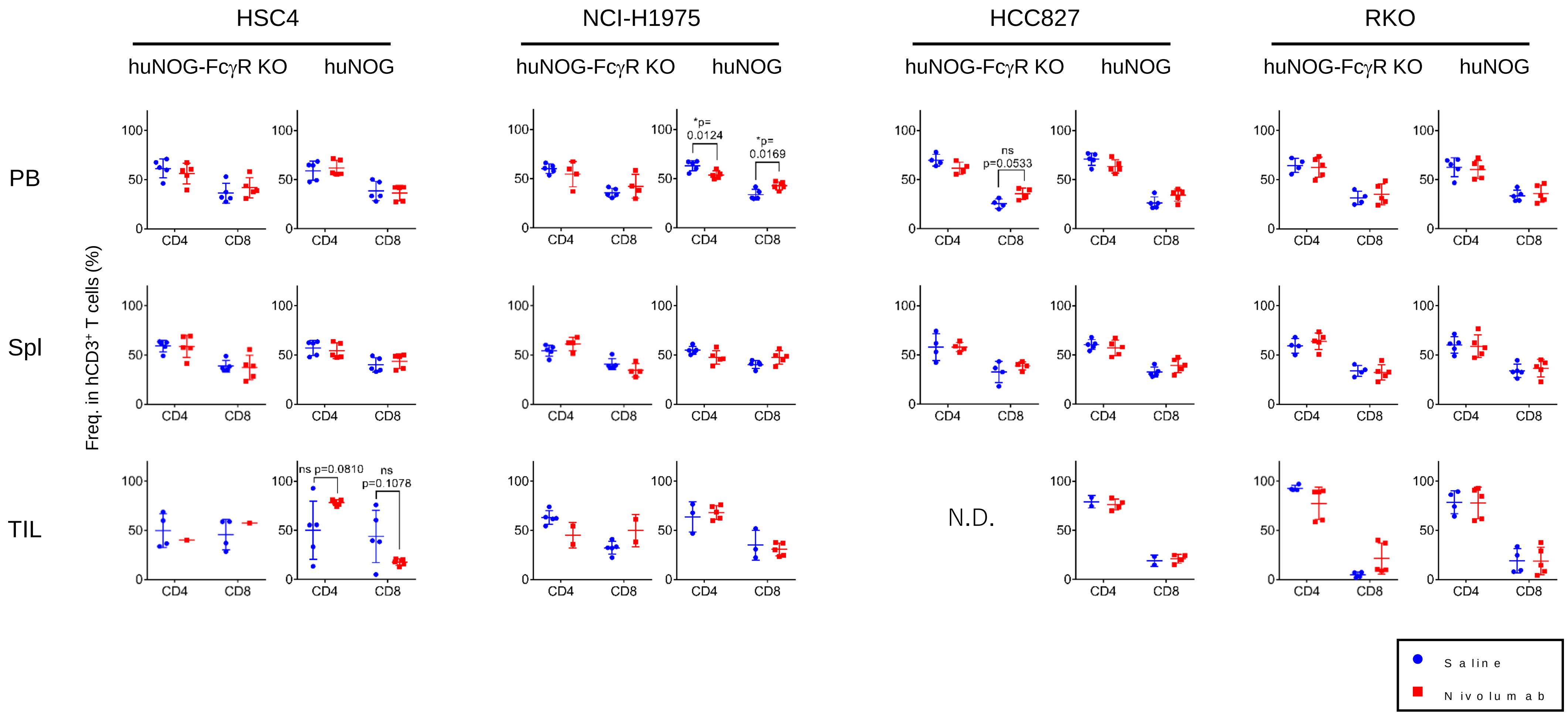

Supplementary Figure 8

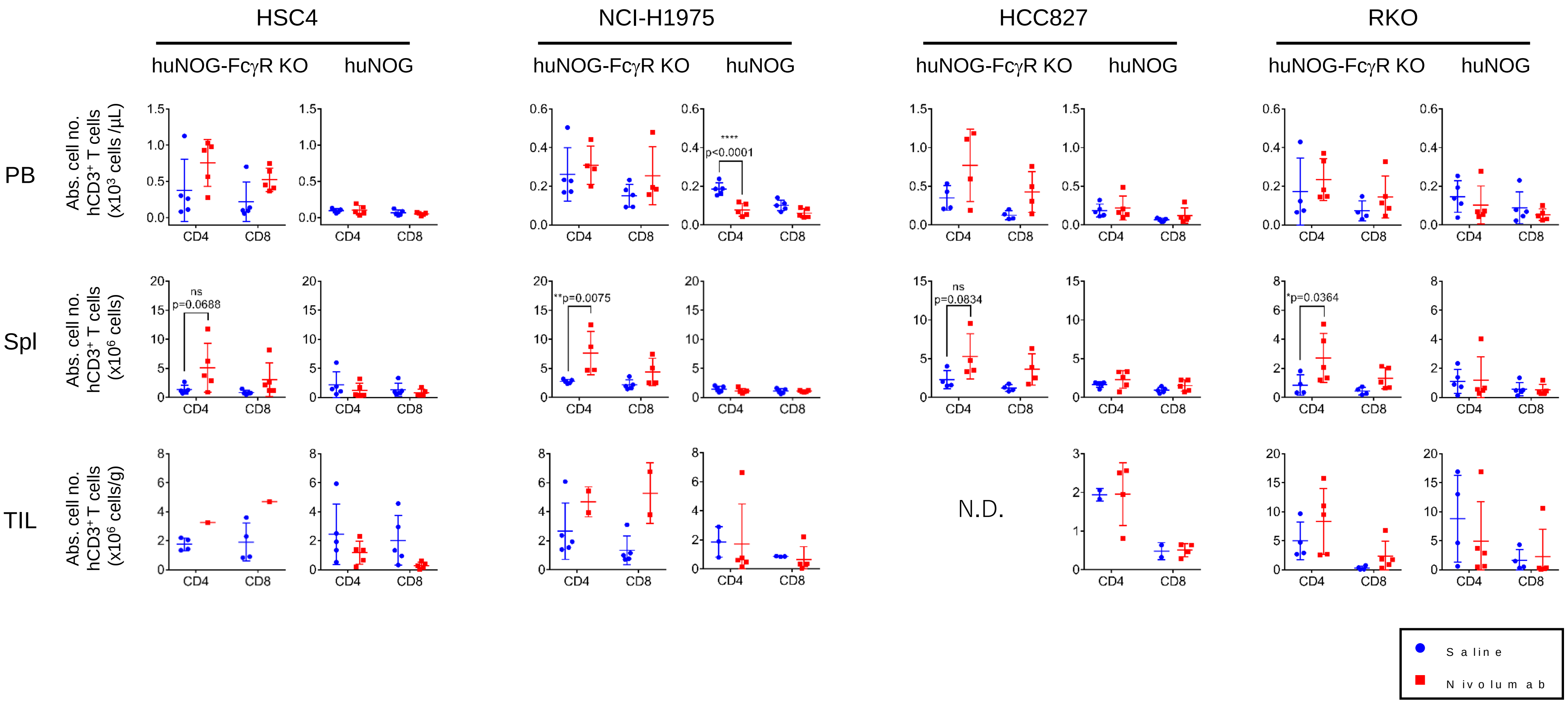

Supplementary Figure 9

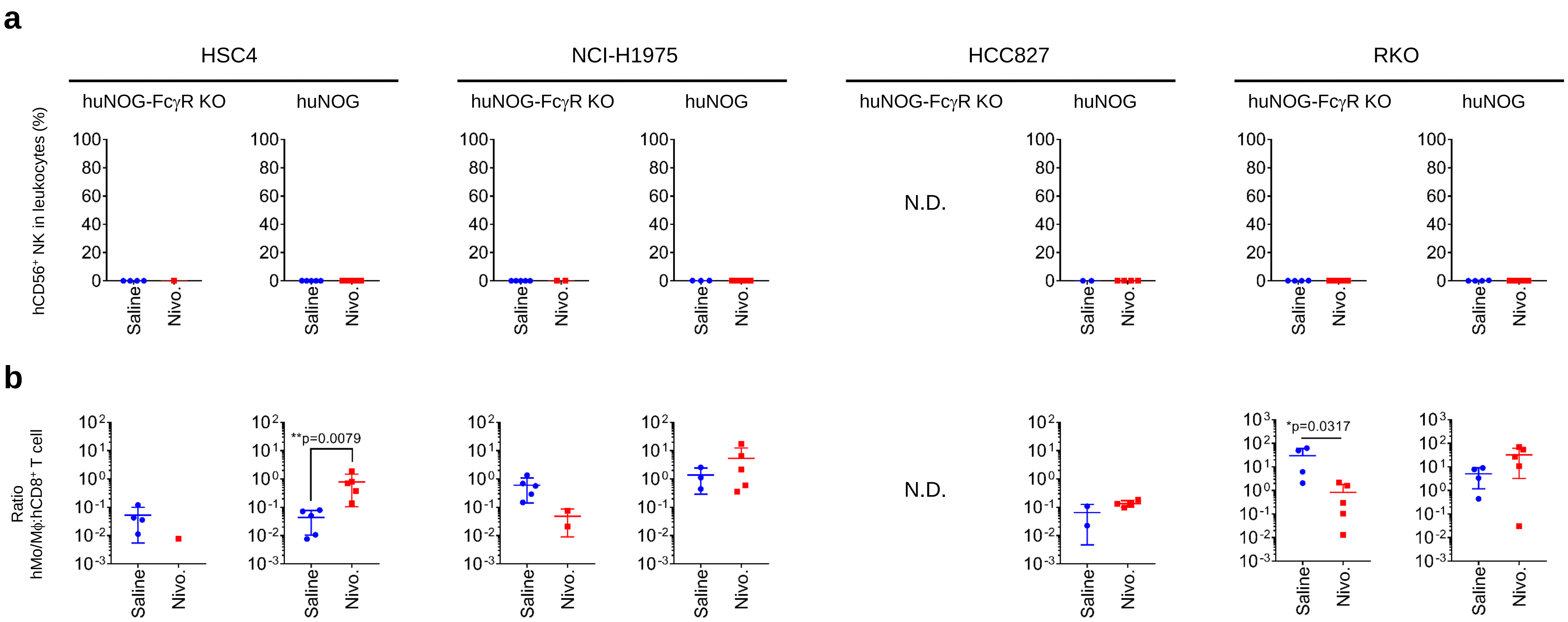

Supplementary Figure 10

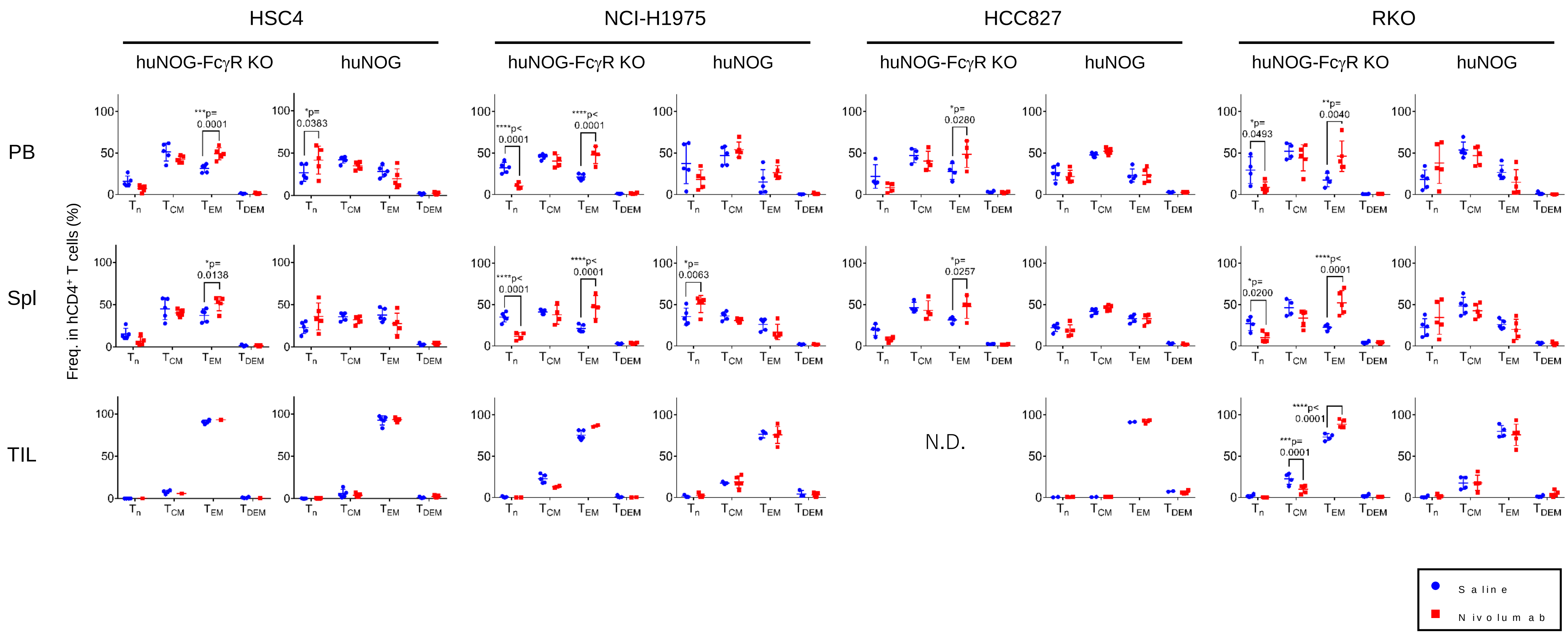

Supplementary Figure 11

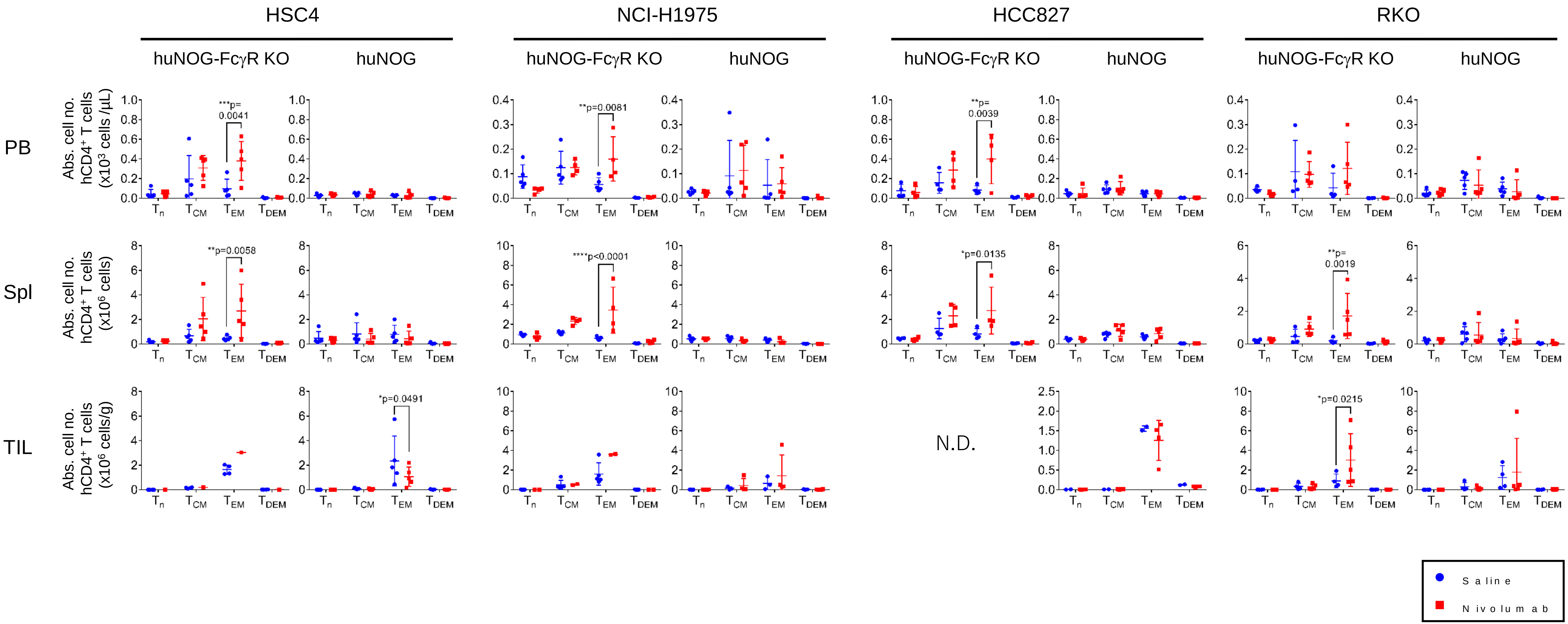

Supplementary Figure 12

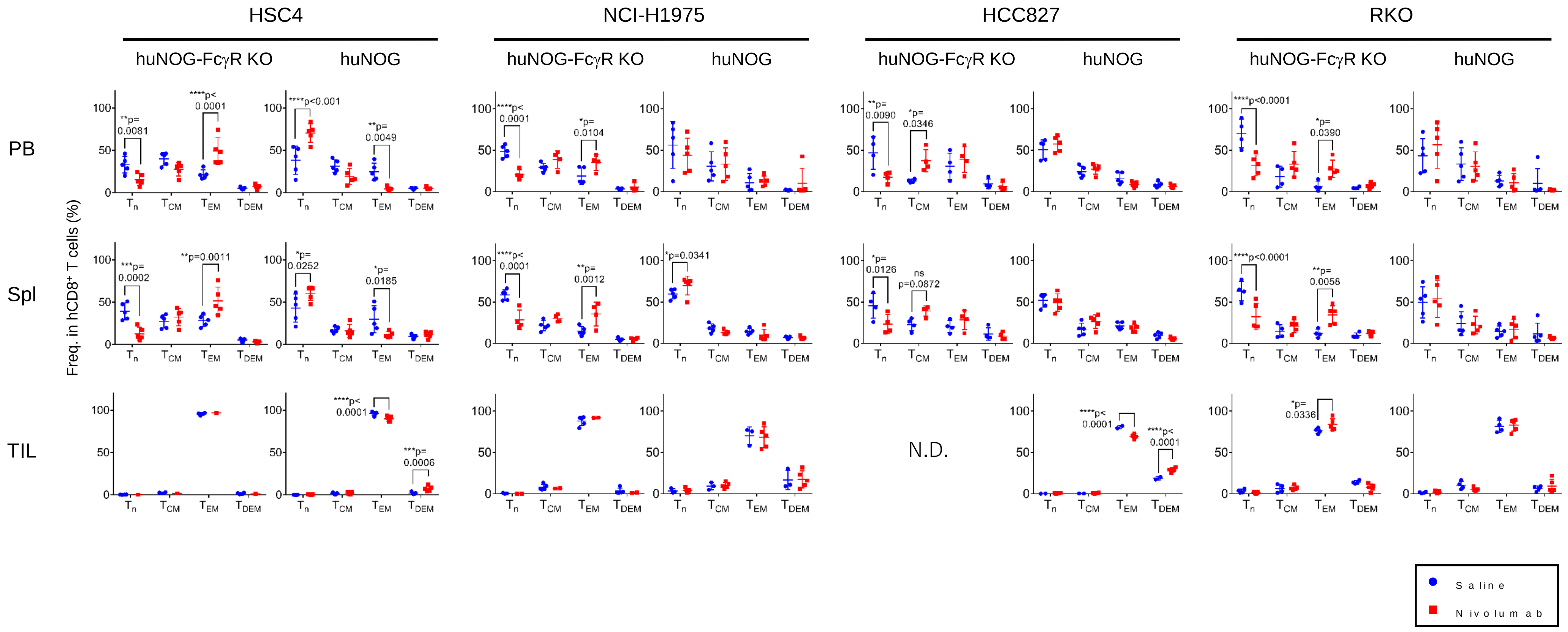

Supplementary Figure 13

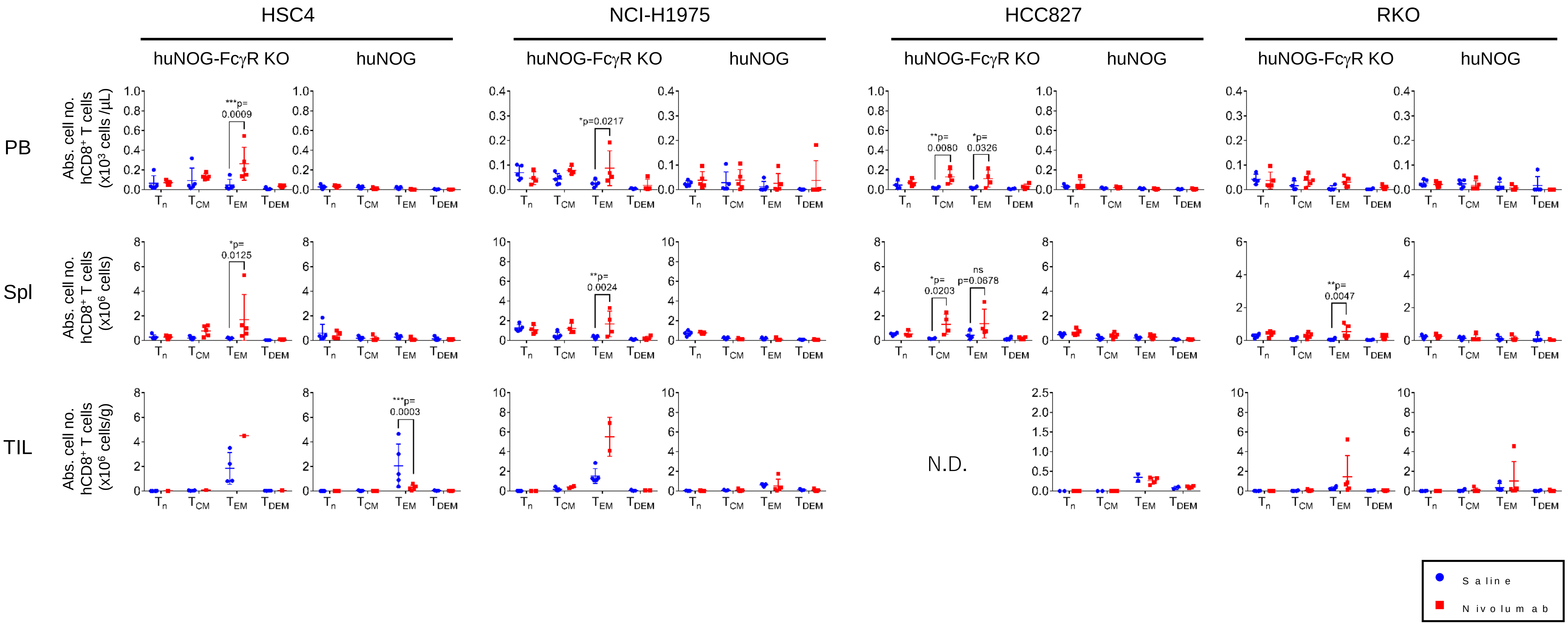

Supplementary Figure 14

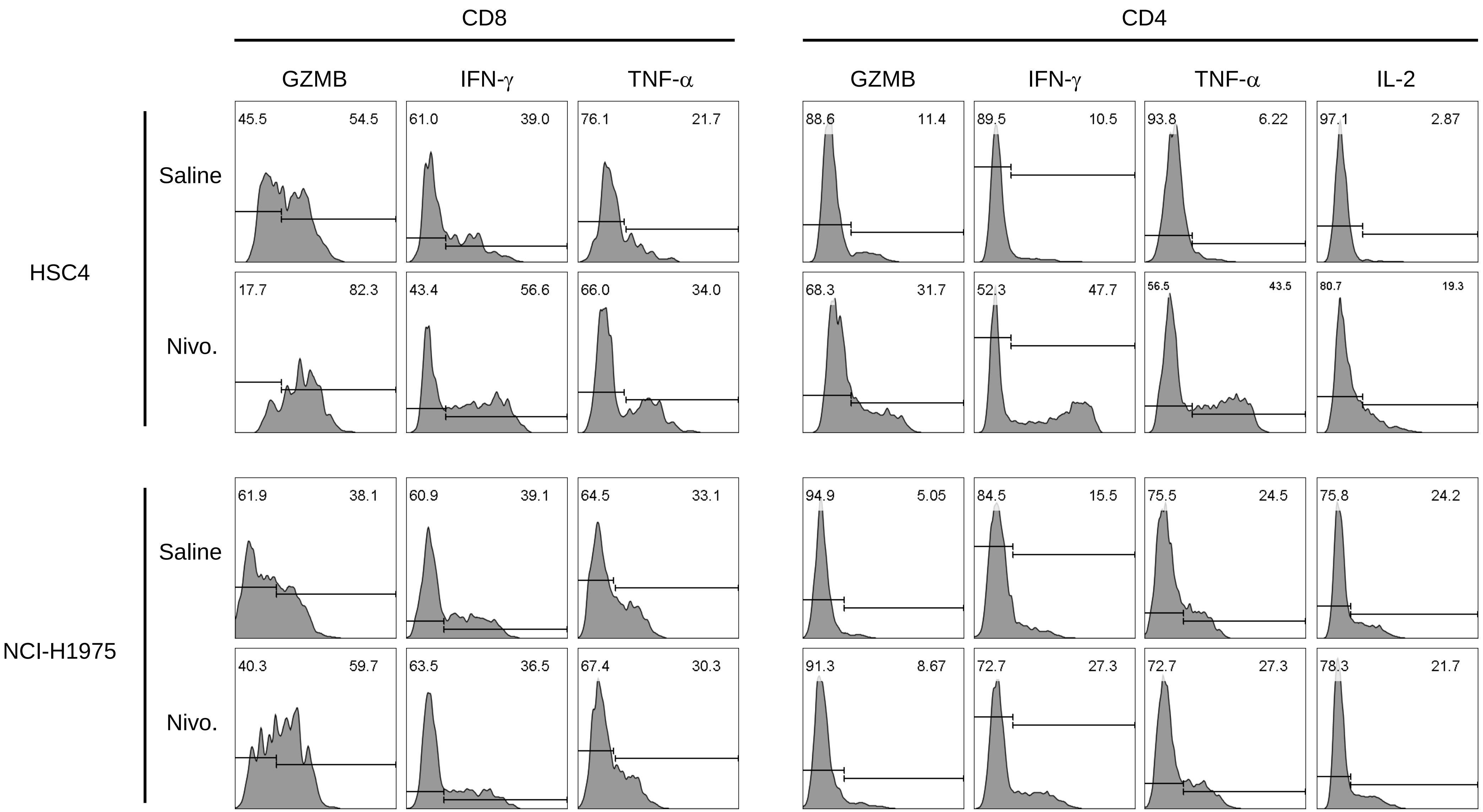

Supplementary Figure 15

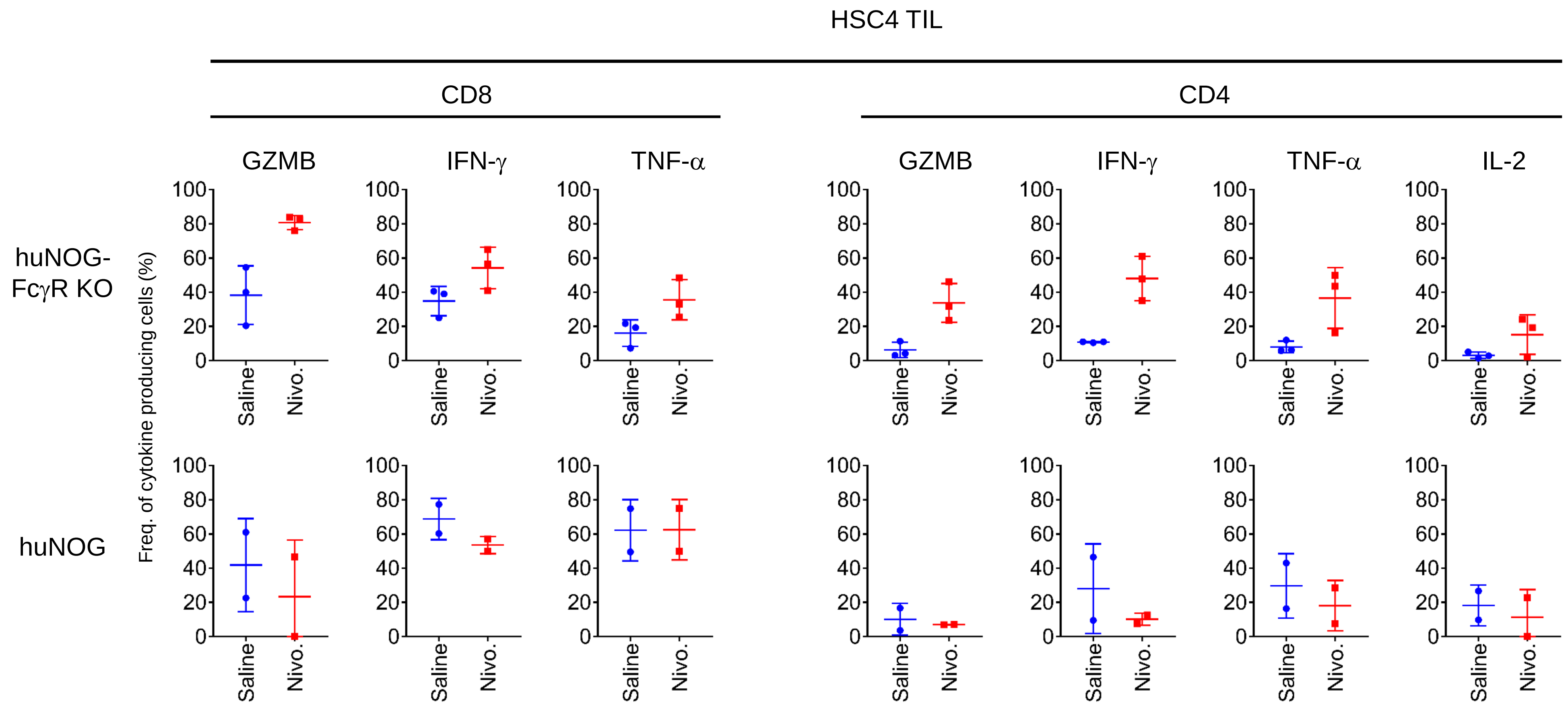

Supplementary Figure 16

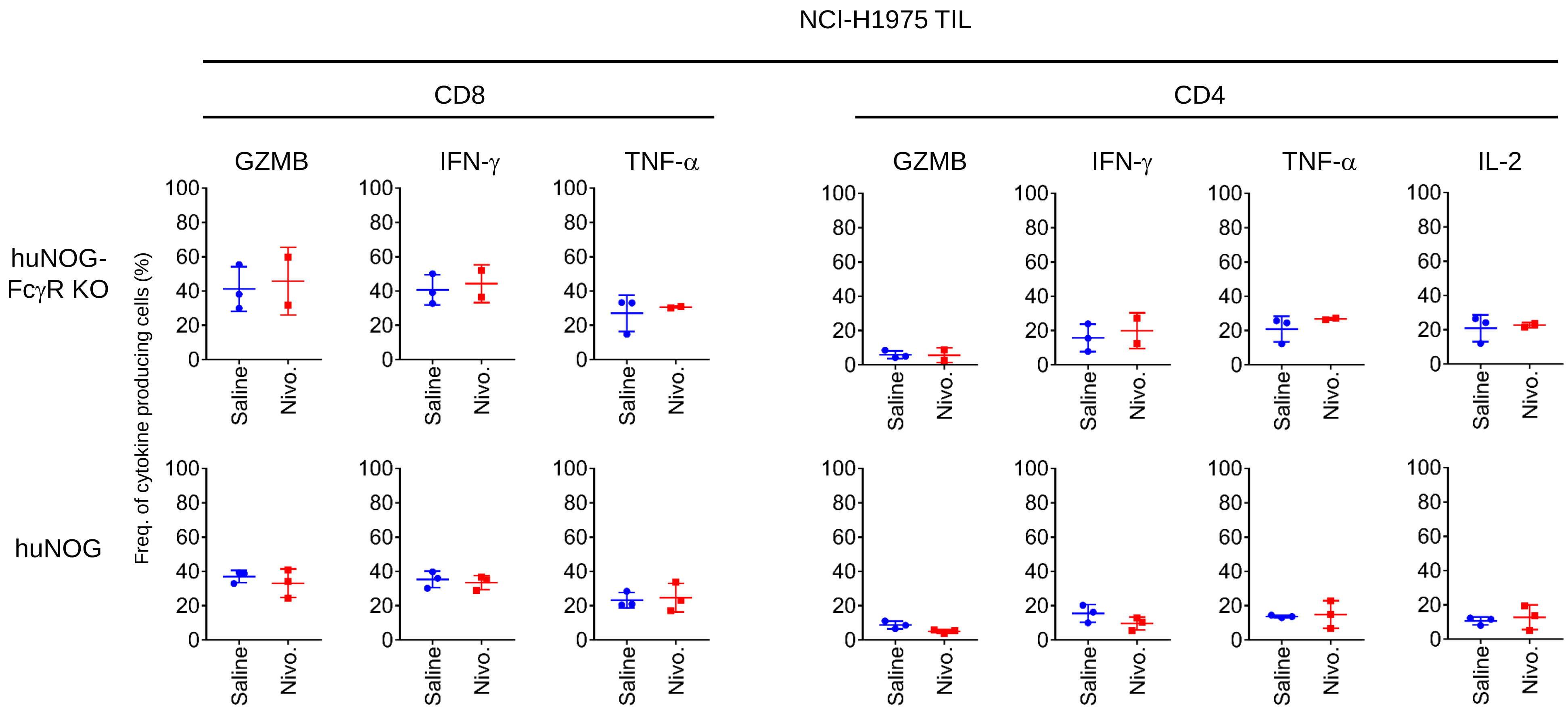

Supplementary Figure 17

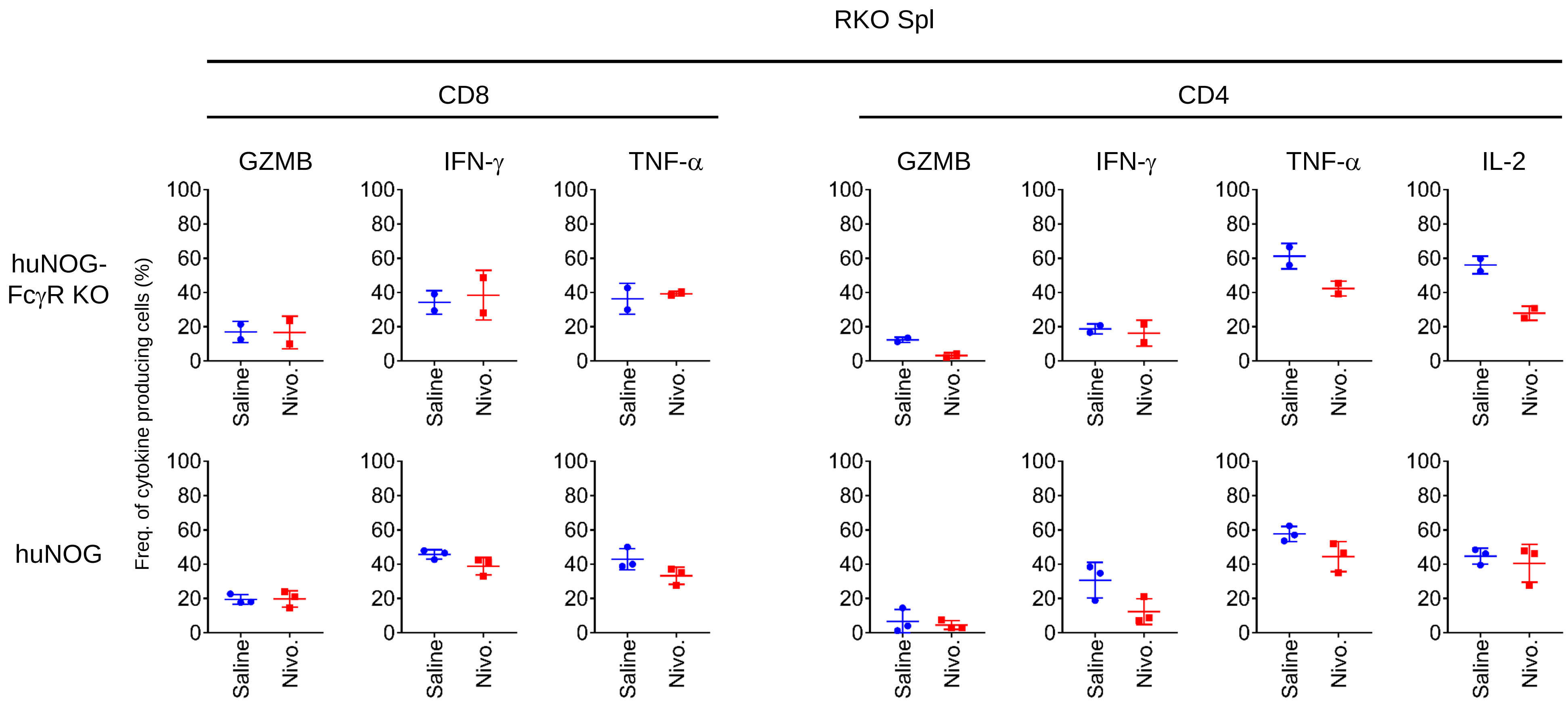

Supplementary Figure 18

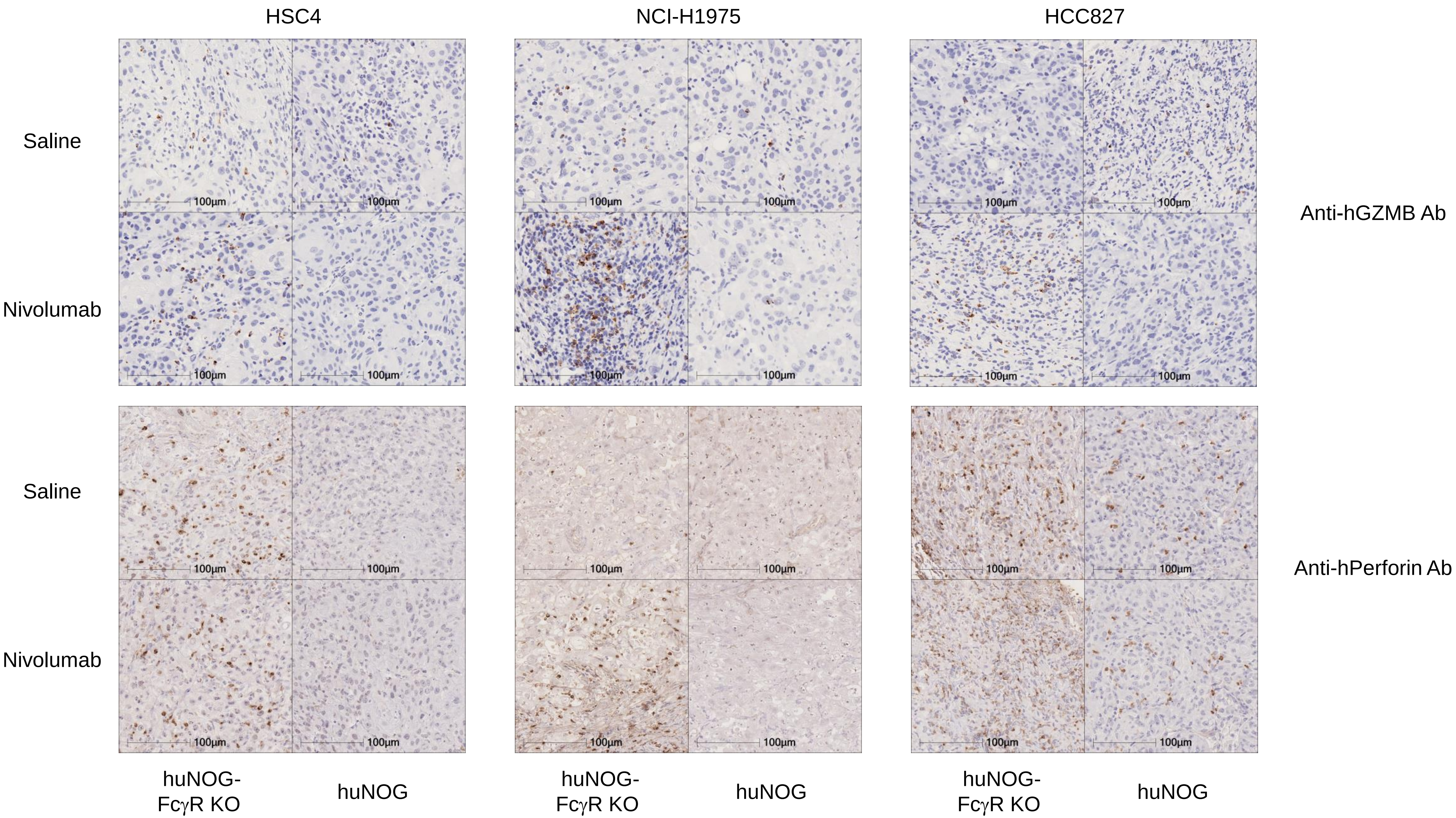

Supplementary Figure 19

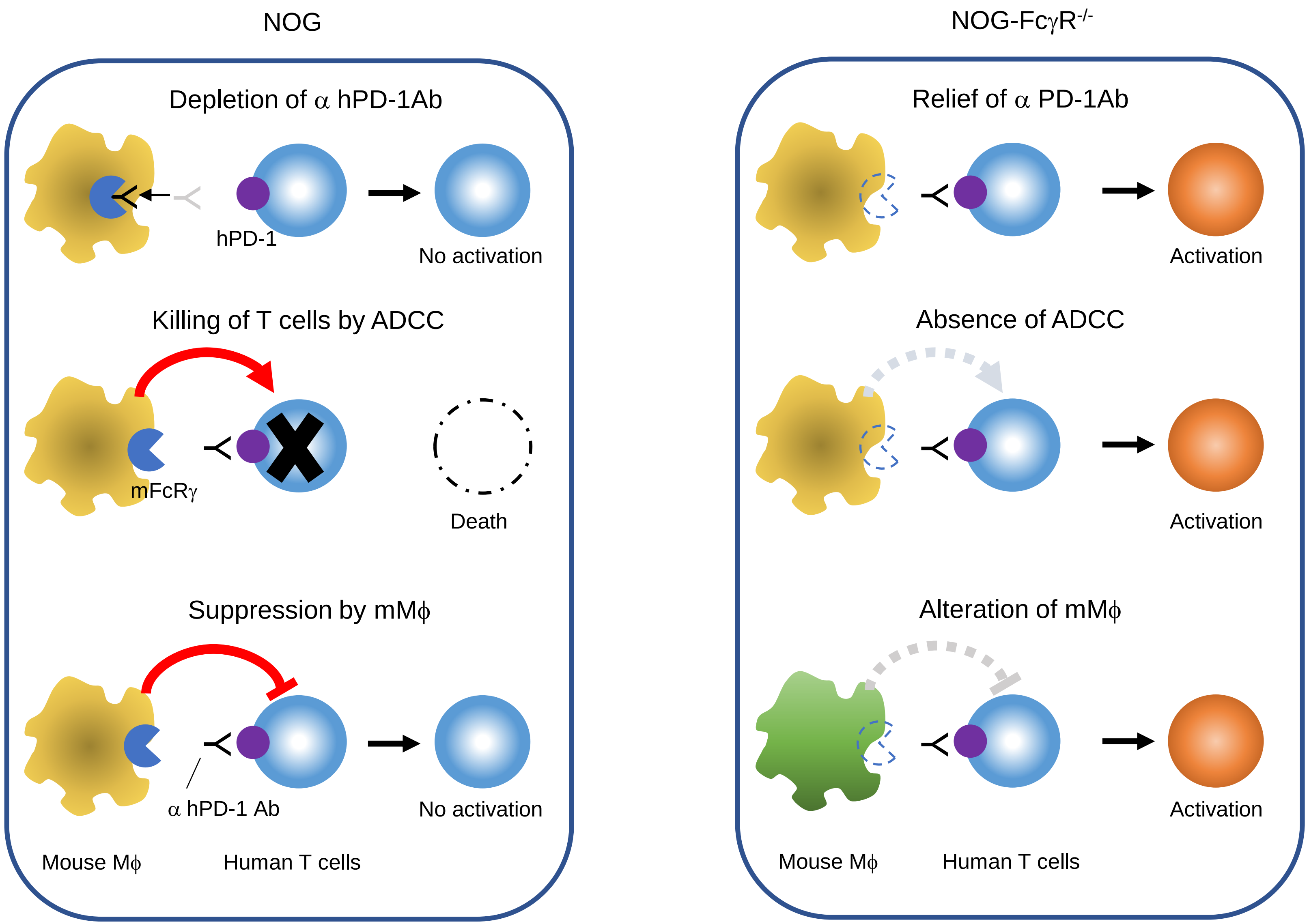

T cell activation by nivolumab in huNOG-FcγR$^{-/-}$ mice
